# Anticitrullinated protein antibodies facilitate migration of synovial tissue-derived fibroblasts

**DOI:** 10.1136/annrheumdis-2018-214967

**Published:** 2019-09-03

**Authors:** Meng Sun, Bence Rethi, Akilan Krishnamurthy, Vijay Joshua, Alexandra Circiumaru, Aase Haj Hensvold, Elena Ossipova, Caroline Grönwall, Yanying Liu, Marianne Engstrom, Sergiu Bogdan Catrina, Johanna Steen, Vivianne Malmstrom, Lars Klareskog, Camilla Svensson, Caroline Ospelt, Heidi Wähämaa, Anca Irinel Catrina

**Affiliations:** 1 Rheumatology Unit, Department of Medicine, Karolinska University Hospital, Karolinska Institutet, Stockholm, Sweden; 2 Department of Rheumatology and Immunology, Peking University People's Hospital, Beijing, China; 3 Molecular Medicine and Surgery, Karolinska University Hospital and Institutet, Stockholm, Sweden; 4 Physiology and Pharmacology, Karolinska Institutet, Stockholm, Sweden; 5 Center of Experimental Rheumatology, Zurich, Switzerland

**Keywords:** Anti-CCP, Rheumatoid Arthritis, Fibroblasts, Autoantibodies, Autoimmune Diseases

## Abstract

**Objectives:**

Rheumatoid arthritis (RA)-specific anti-citrullinated protein/peptide antibodies (ACPAs) might contribute to bone loss and arthralgia before the onset of joint inflammation. We aimed to dissect additional mechanisms by which ACPAs might contribute to development of joint pathology.

**Methods:**

Fibroblast-like synoviocytes (FLS) were isolated from the synovial membrane of patients with RA. The FLS cultures were stimulated with polyclonal ACPAs (anti-CCP-2 antibodies) purified from the peripheral blood of patients with RA or with monoclonal ACPAs derived from single synovial fluid B cells. We analysed how ACPAs modulate FLS by measuring cell adhesion and mobility as well as cytokine production. Expression of protein arginine deiminase (PAD) enzymes and protein citrullination were analysed by immunofluorescence, and signal transduction was studied using immunoblotting.

**Results:**

Challenge of FLS by starvation-induced stress or by exposure to the chemokine interleukin-8 was essential to sensitise the cells to ACPAs. These challenges led to an increased PAD expression and protein citrullination and an ACPA-mediated induction of FLS migration through a mechanism involving phosphoinositide 3-kinase activation. Inhibition of the PAD enzymes or competition with soluble citrullinated proteins or peptides completely abolished the ACPA-induced FLS migration. Different monoclonal ACPAs triggered distinct cellular effects in either fibroblasts or osteoclasts, suggesting unique roles for individual ACPA clones in disease pathogenesis.

**Conclusion:**

We propose that transient synovial insults in the presence of a certain pre-existing ACPA repertoire might result in an ACPA-mediated increase of FLS migration.

**Key messages**

**What is already known about this subject?**

- Anticitrullinated protein/peptide antibodies (ACPAs) exist prior to the onset of rheumatoid arthritis (RA), however, it is unclear how autoimmunity in some but not all cases translate into manifest joint inflammation.

**What does this study add?**

- Cellular stress and pro-inflammatory mediators (interleukin-8) can sensitise synovial fibroblasts to ACPAs by enhancing protein arginine deiminase enzyme expression and cellular citrullination.
- ACPAs promote synovial fibroblast migration through a phosphoinositide 3-kinase-mediated mechanism.
- Different monoclonal ACPAs have distinct cellular effects with three clones increasing migration of challenged fibroblasts, with no effect on osteoclasts and another clone increasing osteoclast differentiation with no effect on fibroblasts.

**How might this impact on clinical practice or future developments?**

- Our results suggest that unique ACPAs may be responsible for specific pathological features in ACPA+RA.
- Inducible protein citrullination could be a key event in the transition of a systemic humoral autoimmunity towards the inflammation of the joints.

## Introduction

Anti-citrullinated protein antibodies (ACPAs) are present in a majority of patients with rheumatoid arthritis (RA) and are specific for this disease.^1^ They consist of a group of antibodies with different specificities towards citrullinated antigens that might cross-react with other protein modifications but not with the native proteins^2–4^ and have been suggested to contribute to joint pain and bone loss already before onset of joint inflammation in RA.^5–8^ In line with this, we and others have shown that polyclonal ACPAs bind to the surface of developing osteoclasts (OC) and suggested that reactivity to citrullinated targets increase OC differentiation and bone loss.^9 10^ Furthermore, experiments in mice have shown that polyclonal ACPAs (defined as anti-CCP-2 IgG antibodies) induces pain-related behaviours even though no joint inflammation develops,^11^ similar to the predisease stage of pain described in ACPA-positive individuals. We originally proposed that this, as well as ACPA-induced bone loss in mice, occurred through an interleukin (IL)-8-dependent and citrulline-specific mechanisms.^10 11^ However, recent papers and corrections^12 13^ this year have led to a reconsideration and extension of the concept. As such also other RA-derived monoclonal antibodies than those with citrulline reactivity and immune complexes are able to cause functional effects similar to those of polyclonal ACPAs, through different mechanisms that are potentially distinct between autoantibody subsets and might include both antigen-driven and Fcγ receptor activation-driven pathways.^14–16^ Taken together, these data suggest a new concept where different RA-associated antibodies with different reactivities contribute to bone loss and pain, potentially through different mechanisms, a complex scenario that requires additional investigations. The need for these investigations and the ways of performing them has been highlighted in a recent editorial.^17^

Previous studies have shown that in the presence of pre-existing joint inflammation in mice, transfer of a monoclonal ACPA may enhance synovial tissue injury,^18^ suggesting that additional local stimuli might be essential for sensitisation of the synovial compartment to effects of antibodies. In the synovial tissue, fibroblast-like synoviocytes (FLS) contribute to an inflammatory stroma that promote and amplify tissue-specific immune activation through the release of various cytokines and have the capacity to grow into the cartilage surface and create an erosive interface by producing tissue remodelling proteases, such as matrix metalloproteinases and cathepsins.^19^ RA-derived FLS have also an increased migration capacity, a feature that might contribute to disease propagation within and in between the joints.^20 21^

In the present report, we investigated ACPA effects on FLS. We demonstrate that stimuli such as cellular stress and/or exposure to pro-inflammatory mediators are needed for the sensitisation of FLS to ACPAs. Furthermore, some but not all ACPAs are able to affect FLS. Our findings thereby suggest that transient synovial insults in the presence of certain pre-existing ACPA clones might act in concert to promote ACPA-mediated FLS migration.

## Materials and methods

Additional detailed information is provided in online supplementary file.

10.1136/annrheumdis-2018-214967.supp1 Supplementary data

### Patient material

Polyclonal ACPA IgGs (anti-CCP2 antibodies) and non-ACPA control IgGs were purified from a pooled serum of patients with RA^22^ and from non-pooled sera of four ACPA-positive patients with RA (for details see online supplementary file). Using a similar method, polyclonal antiproteinase 3 (PR3) IgGs and non-anti-PR3 control IgGs were purified from the sera of a patient with PR3-associated vasculitis. Monoclonal antibodies were isolated from patients with RA as previously described^2 4 15^ and recombinantly expressed as IgG1. All IgG underwent extensive quality control testing including specificity evaluation, size exclusion chromatography aggregation test and limulus amebocyte lysate (LAL) endotoxin test.^23^ F(ab’)2 antibody fragments were generated and RF IgM antibody was purchased from Athens Research & Technology (Georgia, USA). RA-derived and osteoarthritis (OA)-derived synovial fibroblasts as well as human dermal fibroblasts were used in cell culture experiments. Synovial biopsies were obtained from four healthy volunteers and four patients with RA. Patients were enrolled at Karolinska University Hospital. Informed consent was obtained according to the protocol approved by the Ethical Review Committee North of Karolinska University Hospital.

### Migration and adhesion assays

Fibroblast migration was assessed by IncuCyte Zoom live cell imaging system or by scratch assay. Adhesion assays were performed using 16-well E-plates and the xCELLigence System Real-Time Cell Analyzer (ACEA Biosciences, USA). When indicated, the cells were washed with phosphate buffered saline and starved for 2 hours in medium without fetal bovine serum. Citrullinated or native human purified fibrinogen, recombinant vimentin, α-enolase and histone H4 were added as pretreatment to ACPA and control IgGs at 4°C overnight. Phosphoinositide 3-kinase (PI3K) was inhibited by wortmanin and phosphatase and tensin homolog (PTEN) by SF-1670 (Sigma-Aldrich, Stockholm, Sweden). PAD activity was inhibited by Cl-amidine (Cayman Chemical, Ann Arbor, Michigan, USA) or GSK199 (kind gift from Aaron Winkler, Pfizer) for 72 hours prior to scratching. Histone acetyltransferase or deacetylase enzymes were inhibited by anacardic acid or trichostatin A (Sigma-Aldrich, Stockholm, Sweden), respectively. Fibroblasts were cultured in the presence or absence of IL-8 (R&D Systems, Abingdon, UK) or tumour necrosis factor (TNF)-α (Peprotech, London, UK). Cytokine levels were analysed using the Cytometric Bead Array (BD Biosciences, San Diego, California, USA) and matrix metalloproteinases were measured by ELISA (R&D Systems, Abingdon, UK).

### Immunohistochemistry and confocal microscopy

FLS were stained with indicated primary antibodies followed by horseradish peroxidase (HRP)-conjugated antibodies and developed by Vectastain Elite ABC and DAB (Vector Laboratories, Peterborough, UK) and then counterstained with Mayer’s haematoxylin. For confocal microscopy, cells and tissues incubated with primary and secondary antibodies and then counterstained with 4,6-diamidino-2-phenylindole.

### Mass spectrometry

Starved and non-starved FLS whole cell lysate were analysed by mass spectrometry for detection of citrullination.

### Western blot analysis

Cells lysates were loaded an SDS-PAGE gel (Bio-Rad, Solna, Sweden), transferred to nitrocellulose membranes and incubated with primary antibodies and peroxidase-conjugated secondary antibodies.

### Flow cytometry

Cells were collected using non-enzymatic detachment reagent (Sigma-Aldrich, Stockholm, Sweden), stained by fluorochrome-labelled antibodies and analysed by FACSVerse flow cytometer (BD Biosciences, San Jose, California, USA).

### Statistical analysis

The data were analysed using either Kurskal-Wallis test followed by Dunn’s multiple comparation test or two-way analysis of variance, followed by Tukey’s post hoc test by using GraphPad Prism 6 software (GraphPad Software, San Diego, California, USA). P values <0.05 were considered to be statistically significant.

## Results

### Polyclonal ACPAs enhance migration and adhesiveness in starved FLS

**Figure 1:**
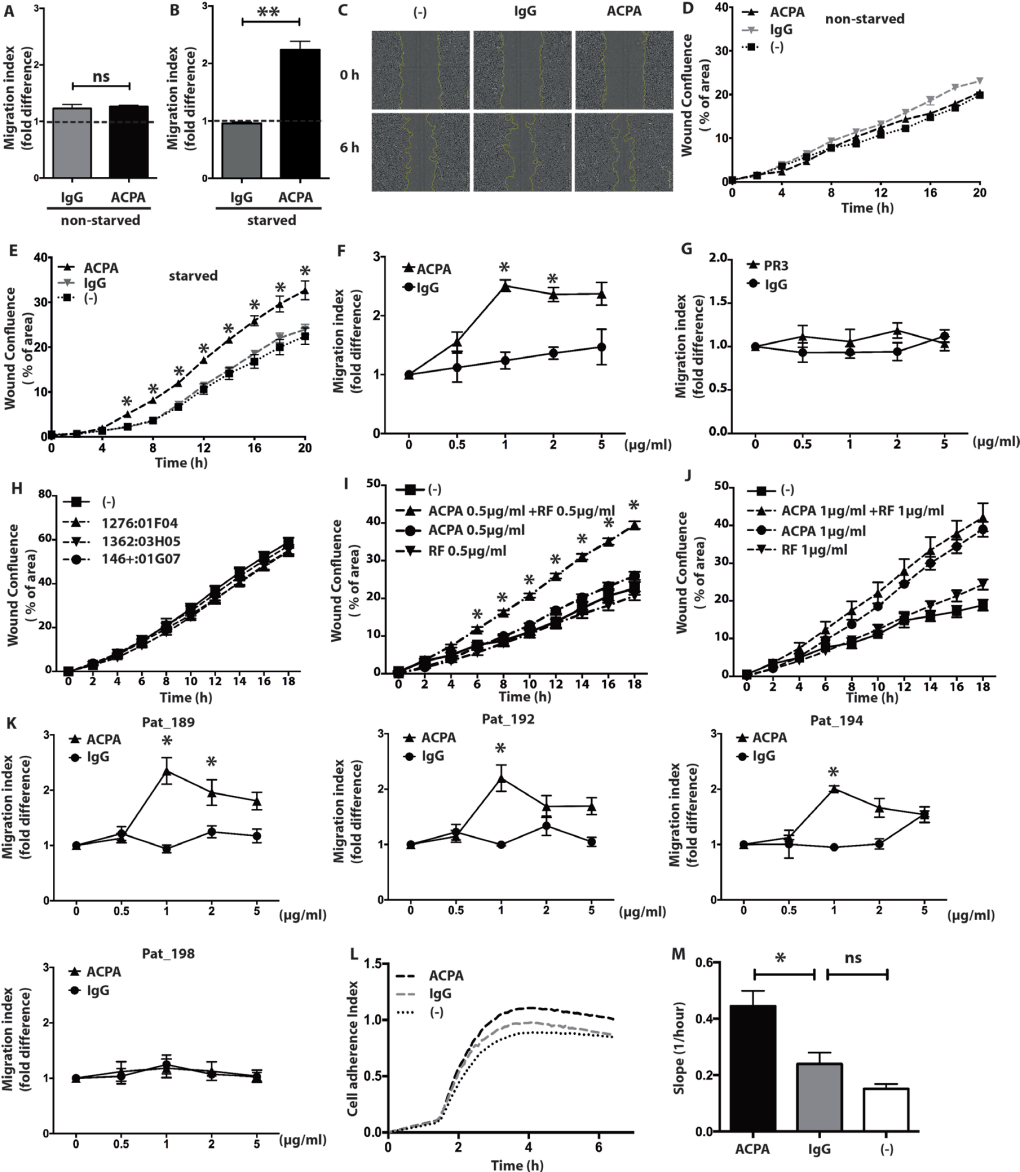
Increased mobility and adherence of synovial fibroblasts in presence of polyclonal ACPAs. Cell mobility was analysed during a period of 6 hours in the presence of 1 µg/mL polyclonal ACPA IgGs (ACPA) or non-ACPA control IgGs (IgG) or without antibody treatment (-) in both non-starved (A) and starved (B) fibroblast cultures. The graphs represent mean±SD values obtained from 10 independent experiments, using cells of 10 different patients and 6 replicates for each treatment. Dot line indicate migration index of non-treated FLS. Image-based evaluation of cell migration in starved fibroblast cultures were performed using phase contrast microscopy with 10x magnification (C). Real-time cell migration was measured in the presence of 1 µg/mL ACPA or control IgG or without any treatment in non-starved (D) and starved (E) fibroblast cultures using IncuCyte. The graphs represent mean±SD values obtained by using cells from 10 individuals patients and 6 replicates in 10 independent experiments. A titration experiment was performed on starved synovial fibroblasts with presence of ACPA (F) and anti-PR3 (G) with indicated concentrations. (H) Cell mobility were tested on starved FLS with presence of 1 µg/mL anti-MDA antibodies (1276:01F04, 1362:03H05 and 146+:01G07). The graphs represent mean±SD values obtained from five replicates in three independent experiments for anti-PR3 titration and anti-MDA antibodies. Starved FLS mobility was analysed by combining 0.5 µg/mL ACPA with 0.5 µg/mL IgM RF (I) and 1 µg/mL ACPA with 1 µg/mL IgM RF (J). The graphs represent mean±SD values obtained from three different patients with six replicates in three independent experiments. Effect of ACPA IgGs fractions prepared from individual blood samples were tested on starved synovial fibroblasts using the indicated concentrations (K). The graphs represent results obtained from cells of three different patients, using six replicates in three independent experiment. Real-time cell adherence was monitored in the presence of 1 µg/mL ACPA, control IgG or without antibody treatment in starved fibroblast cultures for up to 6 hours. The chart shows representative results in one experiment (L), whereas the graph indicates mean±SD values of slope during real-time cell adherence assay, calculated from five different patients with three replicates in five independent experiments (M). *P<0.05. ACPA, anticitrullinated protein/peptide antibody; FLS, fibroblast-like synoviocytes; PR3, polyclonal antiproteinase 3; ns, not significant; RF, rheumatoid factor.

To test if FLS are or might be rendered sensitive to ACPAs, we compared the effects of polyclonal ACPA and non-ACPA control IgGs on FLS migration in both basal conditions and after challenging by a 2-hour serum starvation. Neither polyclonal ACPA IgGs nor non-ACPA control IgGs had any effect on the migration of non-starved FLS (figure 1A). In contrast, polyclonal ACPA IgGs, but not non-ACPA control IgGs, increased the migration index of starved FLS with a mean±SD fold of 2.6±0.3 for ACPA IgGs as compared with 0.95±0.2 for non-ACPA control IgGs, p<0.05 (figure 1B,C). ACPA IgGs had no effect on cell viability and proliferation (online supplementary figure 1). Similar results were seen for OA-derived synovial fibroblasts and human dermal fibroblasts (online supplementary figure 2). The increase in cell mobility was observed as early as 6 hours and was stable up to 20 hours after ACPA exposure (figure 1D,E). Dose titration showed an optimal effect at 1 µg/mL (figure 1F). In contrast, polyclonal anti-PR3 autoantibodies prepared in a similar way as ACPA showed no effect on starved FLS migration at doses as high as 5 µg/mL (figure 1G). Furthermore, monoclonal anti-malondialdehyde(MDA) antibodies obtained through the same methodology as the monoclonal ACPAs had no effect on FLS migration (figure 1H). Interestingly, addition of IgM RF to the cultures significantly potentiated the effect of suboptimal (figure 1I) but not higher concentrations of the polyclonal ACPAs (figure 1J), while showing no effect when used alone (at doses as high as 5 µg/mL, online supplementary figure 3). Some but not all ACPA IgG fractions prepared from individual blood samples were able to recapitulate the effect observed with the ACPA IgGs prepared from pooled blood samples (figure 1K).

10.1136/annrheumdis-2018-214967.supp2 Supplementary data

10.1136/annrheumdis-2018-214967.supp3 Supplementary data

10.1136/annrheumdis-2018-214967.supp4 Supplementary data

Besides migration, ACPA IgGs were able to increase adhesiveness of starved FLS (figure 1L), with an average slope increase of 0.44±0.04 for polyclonal ACPA IgGs as compared with 0.24±0.05 for non-ACPA control IgGs, p<0.05 (figure 1M). Notably, ACPAs did not influence either matrix invasion (data not shown), matrix metalloproteinases production or cytokine and chemokine levels (online supplementary figure 4).

10.1136/annrheumdis-2018-214967.supp5 Supplementary data

### Inducible protein citrullination is essential for the ACPAs effect on FLS migration

**Figure 2:**
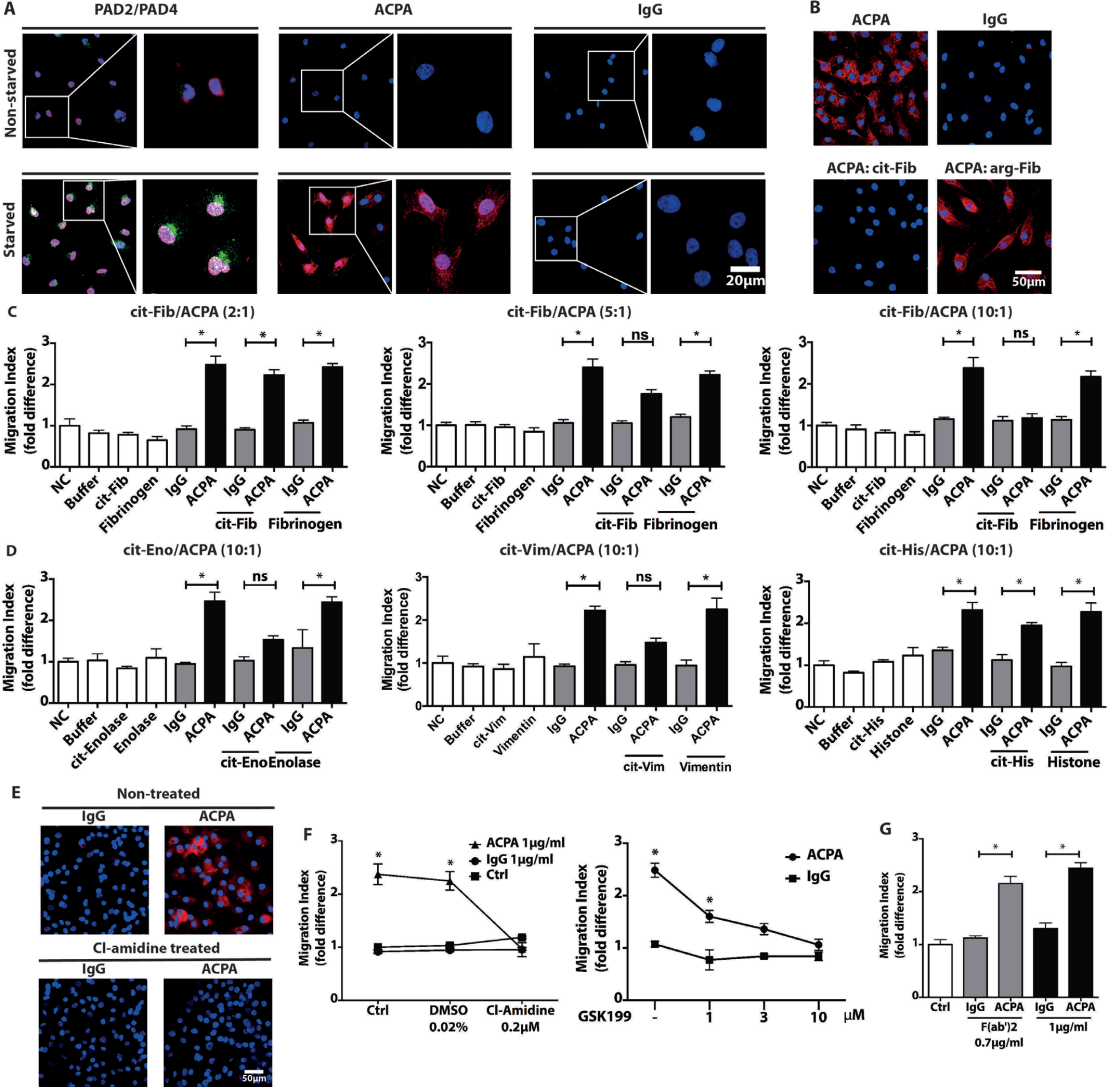
Starvation-induced protein citrullination is essential for synovial fibroblast migration induced by ACPAs. Confocal microscopy images visualise PAD-2 (green) and PAD-4 (red) expressions as well as polyclonal ACPA IgGs (ACPA) and non-ACPA control IgG (IgG) binding patterns in FLS cultures following serum withdrawal (starved) or in non-starved FLS, in the presence of 10% fetal bovine serum (A). An original magnification of 400x was used for all images obtained with confocal microscopy and nuclei are shown in blue. Starved FLS cultures were also stained with ACPAs (red colour) pre-incubated overnight with citrullinated (cit) or native fibrinogen with ratio of protein:antibody (10:1) (cit-Fib or Arg-Fib, respectively) or in the absence of fibrinogen (B). To block ACPA-induced migration, FLS migration was compared in the presence of 1 µg/mL polyclonal ACPAs and control IgG or with both antibodies pre-incubated with citrullinated or native fibrinogen in the indicated ratio overnight. Migration index was calculated from three independent experiments, using cells of three individual patients and six replicates for each treatment, mean±SD values are shown (C). Similar blocking experiments were performed with citrullinated vimentin (Vim), enolase (Eno) and histone 4 (His) using 10:1 decoy protein:antibody ratio. The graphs represent results obtained from three independent experiments, using cells of three individual patients and six replicates (D). To inhibit PAD enzymes, we exposed FLS cultures to 200 nM Cl-amidine for 72 hours. Confocal microscopy images show the inhibition of ACPA binding (red colour) to Cl-amidine pretreated FLS (E). Moreover, after pre-incubation of the cells with different PAD inhibitors, Cl-amidine and GSK199, we could not detect an increased FLS mobility in the presence of ACPAs (F). Migration index was calculated from three independent experiments, using cells of three individual patients and six sample-replicates, mean±SD values are shown. Furthermore, FLS migration were analysed in the presence of 0.7 µg/mL polyclonal ACPA F(ab’)2 and non-ACPA IgG F(ab’)2. The graph indicates results obtained from three independent experiments, using cells of three individual patients and six replicates (G). *P<0.05. ACPA, anticitrullinated protein/peptide antibody; DMSO, dimethyl sulfoxide; FLS, fibroblast-like synoviocytes; PAD, protein arginine deiminase; ns, not significant.

To investigate the mechanisms that render FLS sensitive to ACPAs, we analysed the expression of PAD-2 and PAD-4, two PAD enzymes known to be present in RA synovium,^24^ and cellular citrullination following serum starvation. Non-starved FLS express low levels of intracellular PAD-2 and PAD-4 and do not bind polyclonal ACPA (figure 2A, upper panel). Cellular starvation significantly increased PAD-2 and PAD-4 enzymes expression in a majority of the cultured cells, as well as the amounts of citrullinated proteins labelled by polyclonal ACPA IgGs (figure 2A, lower panel and online supplementary figure 5). This was confirmed by mass spectrometry showing an increase in the number and the amount of citrullinated targets (estimated by label-free quantitative analysis) in starved as compared with non-starved FLS (online supplementary figure 6). Pre-incubation of the polyclonal ACPA with citrullinated (cit) but not native fibrinogen abolished FLS staining by ACPAs (figure 2B), as well as the ACPA-induced migration of starved FLS, an effect that titrated out at a protein:antibody ratio of 2:1 (figure 2C). Additional experiments demonstrated a similar effect for cit-vimentin and cit-enolase but not for cit-histone 4 (figure 2D). In line with this, inhibition of cellular citrullination by pretreatment with PAD inhibitors prevented both ACPA-staining (figure 2E) and the ACPA-induced migration of starved FLS (figure 2F), without affecting proliferation and cell viability (online supplementary figure 1). Furthermore, F(ab’)2 ACPA fragments had a similar capacity to induce migration of starved FLS (figure 2G), in line with lack of expression of Fcγ receptors by FLS (online supplementary figure 7).

10.1136/annrheumdis-2018-214967.supp6 Supplementary data

10.1136/annrheumdis-2018-214967.supp7 Supplementary data

10.1136/annrheumdis-2018-214967.supp8 Supplementary data

**PI3K activation** **is required for** **ACPA-induced FLS migration**

**Figure 3:**
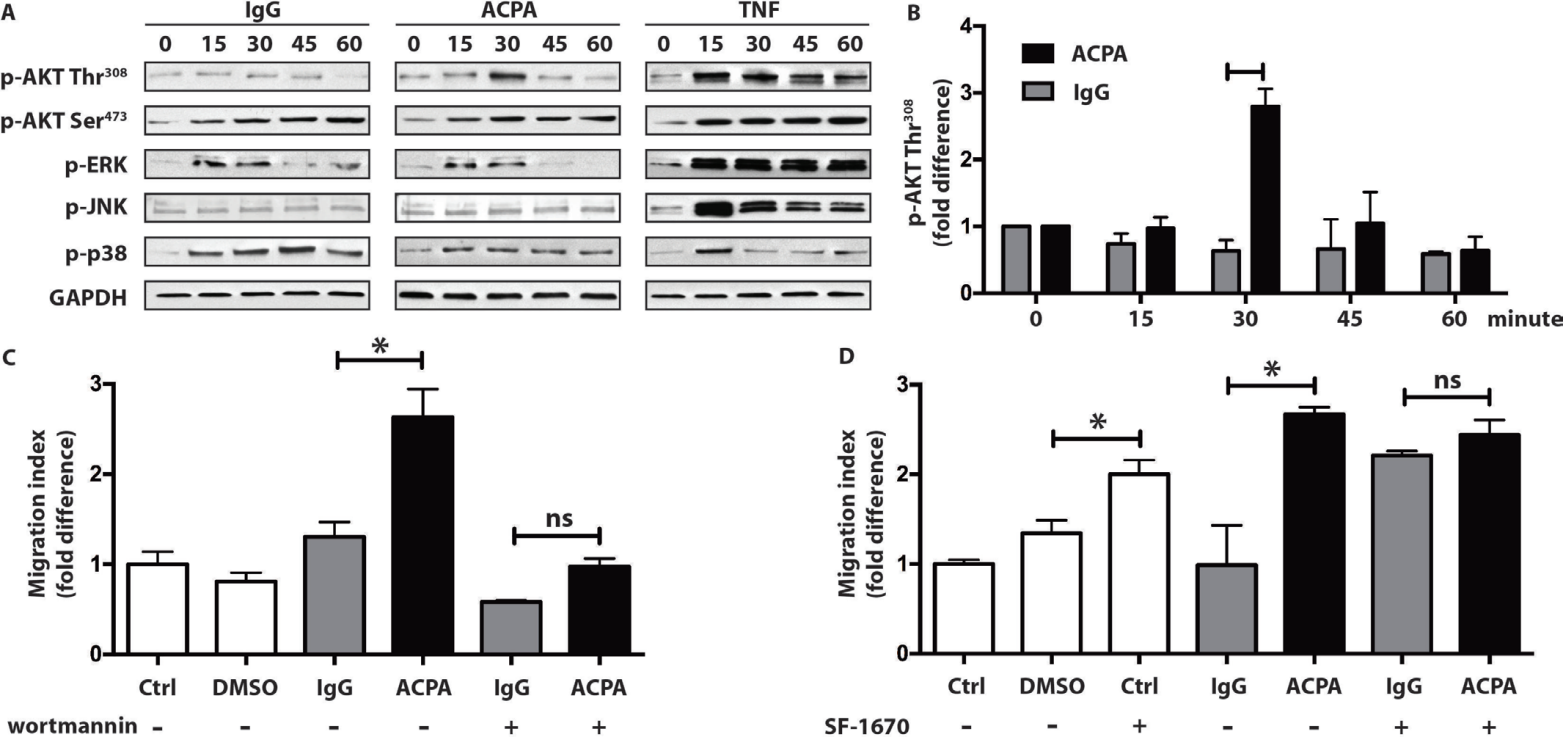
Role of PI3K in the ACPA-induce synovial fibroblast migration. Starved FLS were exposed to 1 µg/mL polyclonal ACPA IgG (ACPA) or non-ACPA control IgG (IgG) or to TNF 10 ng/mL. Immunoblot analysis of Akt (Thr308 or Ser473 residues), ERK1/2, JNK and p38 phosphorylation are shown, whereas GAPDH protein was detected as control. The presented data are representative for at least three independent experiments for all tested proteins (A). Change of Akt phosphorylation at the Thr308 residue is shown in response to 1 µg/mL ACPA or IgG treatments (B). The data were calculated following densitometry analysis in five independent experiments, levels were normalised to baseline intensities, mean±SD values are shown. FLS were pretreated with 200 nM of the PI3K inhibitor wortmannin (C) or 2 µg/mL of the PTEN inhibitor SF-1670 (D) for 2 hours prior to the analysis of cell migration in the presence of 1 µg/mL ACPA or control IgG. DMSO (0.01%) was used as solvent control. Graphs represent fold-change in cell mobility, mean±SD values were calculated from three independent experiments, *P<0.05. ACPA, anticitrullinated protein/peptide antibody; DMSO, dimethyl sulfoxide; FLS, fibroblast-like synoviocytes; PAD, protein arginine deiminase; PI3K, phosphoinositide 3-kinase; PTEN, phosphatase and tensin homolog; ns, not significant; TNF, tumour necrosis factor.

To gain insight into the intracellular signals activated by ACPAs, we investigated two major regulators of FLS migration: PI3K and mitogen-activated protein kinases (MAPK). Polyclonal ACPAs induced a transient but robust peak in Akt phosphorylation at the Thr308 residue linked to PI3K activation (figure 3A left and middle panels and figure 3B), whereas Akt phosphorylation at the Ser473 residue, linked to mammalian target of rapamycin activation, seemed to occur irrespectively of the presence of ACPAs (figure 3A left and middle panels). MAPK phosphorylation showed no differences between polyclonal ACPA and control IgGs. In contrast to the rather selective modulation of Akt by ACPAs, control treatment with TNF-α-induced prolonged and broad phosphorylation events (figure 3A, right panels). PI3K inhibition almost completely abolished the ACPA-induced migration of starved FLS (figure 3C). Furthermore, increased stimulation of PI3K by blocking PTEN led to higher cellular mobility with no further increment by ACPAs (figure 3D).

### Inflammatory stimuli can substitute starvation and sensitise FLS to ACPAs

**Figure 4:**
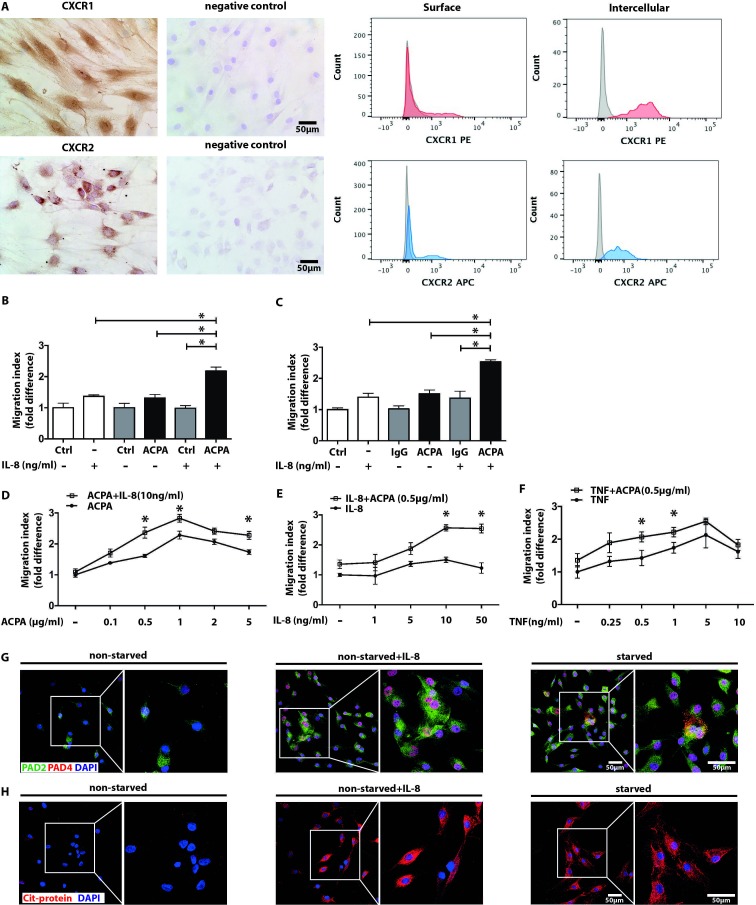
Collaboration of ACPA-mediated signals with the inflammatory cytokines IL-8 and TNF in inducing FLS migration. CXCR1 and CXCR2 expressions in FLS cultures were analysed with immunohistochemistry by light microscopy with the original magnification of 250x (left panel) and flow cytometry (right panel) (A). Migration of non-starved (B) and starved FLS (C) were assessed after exposing the cells to suboptimal (0.5 µg/mL) dose of ACPA or IgG in the presence or absence of 10 ng/mL recombinant human IL-8. Mean±SD values were calculated from three independent experiments, using cells of five individual patients and three replicates, *p<0.05. Starved FLS mobility was analysed by combining high dose (10 ng/mL) of IL-8 with increasing ACPA concentrations (D) or suboptimal concentration of ACPA with increasing concentrations of IL-8 (E). On similar grounds, increasing concentrations of TNF were combined with ACPA applied at a suboptimal dose (F). Mean±SD values were calculated based on at three independent experiments, using cells of three different patients and six sample replicates, *p<0.05 (G). Confocal microscopy images show increased PAD-4 (red colour) and PAD-2 (green colour) expression of non-starved FLS cultures stimulated by IL-8 (10 ng/mL). Nuclei are represented in blue colour. The effect of IL-8 (10 ng/mL) was also tested on ACPA binding to FLS cultures using confocal microscopy (H). Red colour represents antibody binding, nuclei are shown in blue, The original magnification was 400x. The right panels represent a zoomed area from the original images. ACPA, anticitrullinated protein/peptide antibody; DAPI, 4,6-diamidino-2-phenylindole; FLS, fibroblast-like synoviocytes; IL, interleukin; PAD, protein arginine deiminase; ns, not significant; SF, synovial fluid; TNF, tumour necrosis factor.

To identify additional factors that may render FLS sensitive to ACPAs, we investigated the potential contribution of pro-inflammatory stimuli, such as IL-8 and TNF-α. FLS express IL-8 receptors CXCR1 and CXCR2 (figure 4A) and produce low levels of IL-8, unaffected by ACPAs (online supplementary figure 4). Exogenously added IL-8 had no effect on the migration of non-starved (figure 4B) or starved (figure 4C) FLS, but significantly potentiated the effect of polyclonal ACPAs in both cultures. The synergistic effects were further demonstrated by combination of different concentrations of ACPAs with a fixed dose of IL-8 (figure 4D) or suboptimal concentration of ACPAs with increasing concentration of IL-8 (figure 4E). TNF-α triggered a significant increase in FLS migration both in the presence or absence of ACPAs, suggesting different mechanisms triggered by these two cytokines (figure 4F). Similar to cell starvation, exogenous IL-8 significantly increased PAD-2 and PAD-4 expression (figure 4G), as well as the amount of citrullinated proteins (figure 4H) in non-starved FLS (see also online supplementary figure 5).

### Different ACPA clones induce FLS migration and osteoclast activation

**Figure 5:**
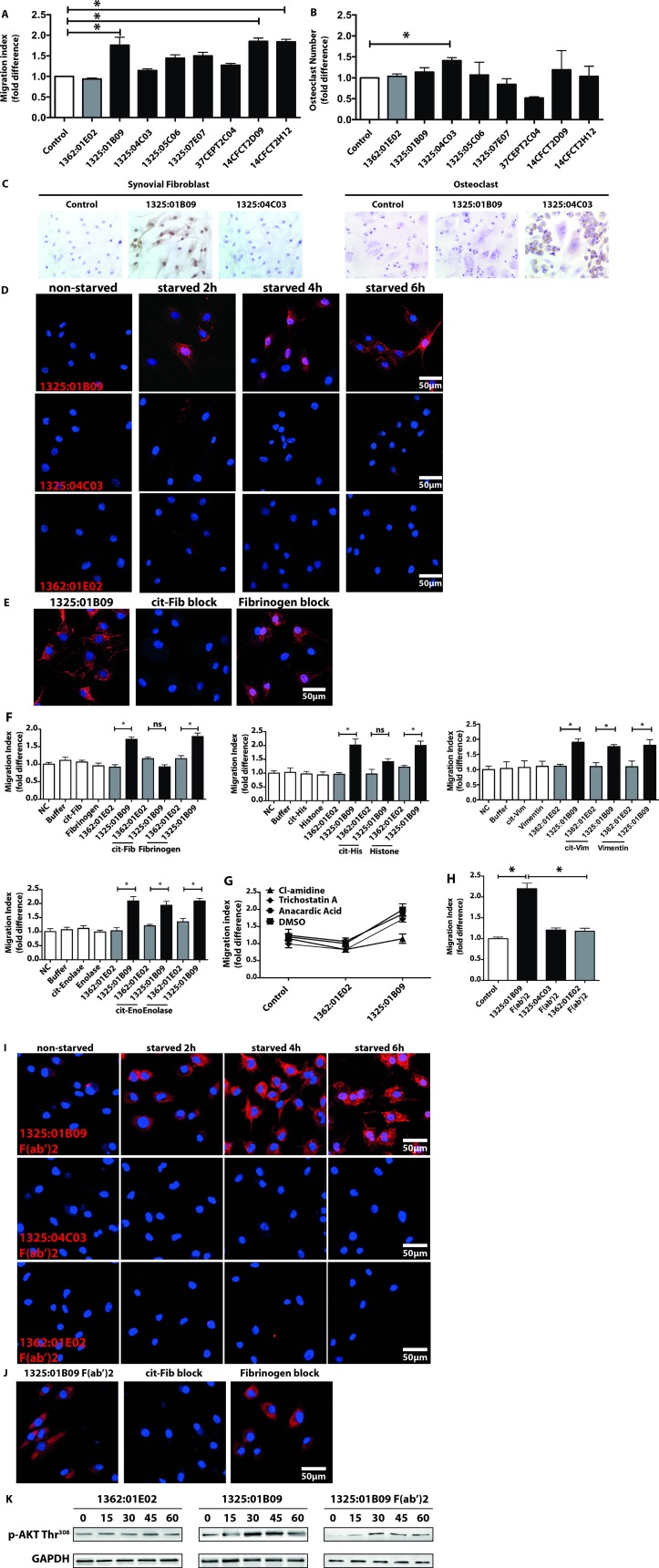
The monoclonal ACPAs 1325:01B09 and 1325:04C03 display selective binding and stimulatory capacities on FLS and osteoclasts. The effects of eight individual ACPA clones were tested in concentration of 1 µg/mL on fibroblast migration (A) and osteoclast differentiation (B) assays. The graphs represent migration index expressed as fold-difference normalised to untreated controls using data from three independent experiments, using cells of three different patients, all performed six replicate samples, *p<0.05. Osteoclast counts expressed in fold-difference normalised to untreated controls using data from two to five independent experiments, performed at least three replicates, *p<0.05. IHC stainings show binding capacity of individual ACPA clones, 1325:01B09 and 1325:04C03 to FLS and osteoclasts (C). Confocal microscopy images illustrate the binding of the ACPA clones (1325:01B09, 1325:04C03) and control (1362:01E02) monoclonal antibodies (red colour) to starved FLS (D). To block monoclonal ACPA binding, starved FLS were also stained with 1325:01B09 (red colour) pre-incubated with citrullinated (cit) or native fibrinogen (cit-Fib or Arg-Fib, respectively) or in the absence of fibrinogen (E). Blocking experiments were performed with cit vimentin (cit-Vim), cit enolase (cit-Eno) and cit histone H4 (cit-His) with 10:1 decoy protein:antibody ratio (F). Migration indexes were obtained from three independent experiments, using cells of three individual patients and six replicates. In addition, monoclonal ACPA-induced migration was blocked by PAD inhibitor (200 nM Cl-amidine), but not by histone acetyltransferase or deacetylase inhibitors (10 µM anacardic acid, 0.2 µM trichostatin A) (G). FLS migration was also analysed in the presence of monoclonal ACPA or control IgG F(ab’)2 fragments at a concentration of 0.7 µg/mL. The graphs represent results from three independent experiments, using cells of three individual patients and six sample-replicates (H). Confocal images illustrate the binding of 1325:01B09, 1325:04C03 and 1362:01E02 F(ab’)2 fragments (red colour) to starved FLS (nuclei shown in blue, origianl magnification 400X (I). Pre-incubation with citrullinated fibrinogen prevented 1325:01B09 F(ab’)2 fragments binding to starved FLS (nuclei shown in blue, original magnification 400X) (J). Nuclei are shown in blue. Original magnifications of 100x and 400x were used in light and confocal microscopy, respectively. Starved FLS were exposed to 1 µg/mL 1325:01B09, 1362:01E02 or 0.7 µg/mL 1325:01B09 F(ab’)2. Immunoblot analysis of Akt phosphorylation (Thr308 residues) is shown, GAPDH protein was detected as control (K). ACPA, anticitrullinated protein/peptide antibody; DMSO,dimethyl sulfoxide; FLS, fibroblast-like synoviocytes; IHC, immunohistochemistry; PAD, protein arginine deiminase; ns, not significant.

Since polyclonal ACPA IgGs contain a wide spectrum of antibodies, we investigated whether monoclonal ACPAs differ in their capacity to affect distinct cellular functions such as FLS mobility and OC differentiation. Among eight monoclonal ACPAs, each with different patterns of antigen recognition, three of them (1325:01B09, 14CFCT2D09 and 14CFCT2H12), enhanced FLS migration significantly (figure 5A, for titration curves see online supplementary figure 8), while not having any effect on OC differentiation (figure 5B). Interestingly, clone 1325:01B09 but not 14CFCT2D09 and 14CFCT2H12 are cross-reactive with homo-citrullinated targets.^4 25^ In contrast, clone 1325:04C03, having no effect on FLS migration (figure 5A), significantly increased osteoclastogenesis (figure 5B) already at a concentration of 1 µg/mL. Selective cellular activation correlated with binding of clone 1325:01B09 to starved FLS and clone 1325:04C03 to OC (figure 5C). Binding of 1325:01B09 to FLS (figure 5D) was prevented by pre-incubation with cit-fibrinogen or cit-histone H4 but not by cit-vimentin or cit-enolase (figure 5E,F). As 1325:01B09 also cross-react with acetylated modified proteins,^25^ we performed further experiments to show that altered citrullination but not acetylation could modulate 1325:01B09 effect on FLS (figure 5G). Furthermore, F(ab’)2 fragments of 1325:01B09 enhanced migration of (figure 5H) and bound to non-permeabilised (figure 5I) challenged FLS which was prevented by exogenously added cit-fibrinogen (figure 5J). Both whole and F(ab’)2 fragments of 1325:01B09 were able to promote migration of challenged FLS through a similar mechanism as for the polyclonal ACPA IgGs, as shown by activation of the PI3K pathway (figure 5K).

10.1136/annrheumdis-2018-214967.supp9 Supplementary data

**Figure 6:**
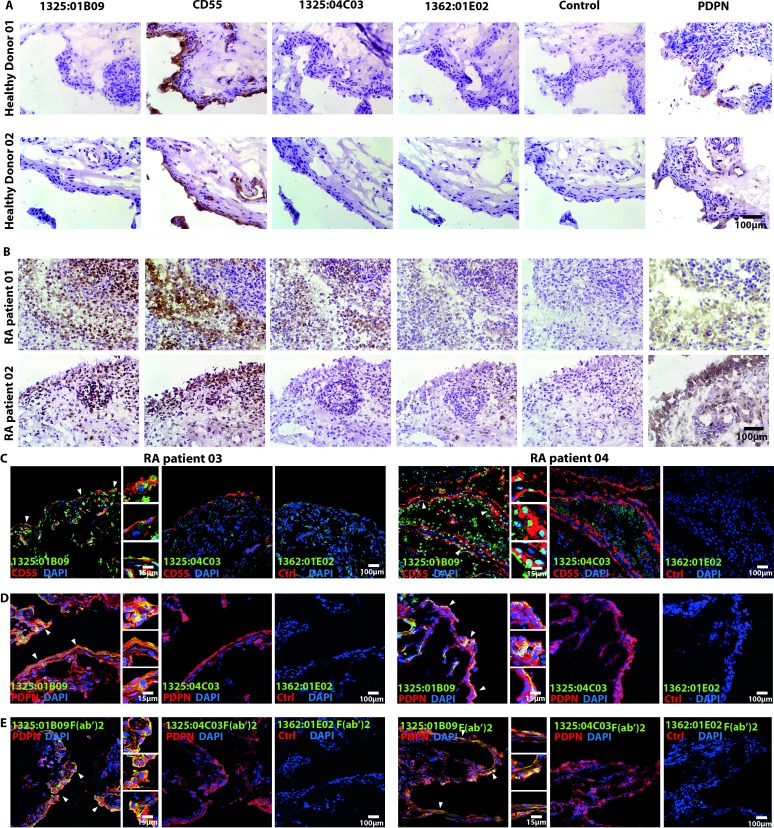
The monoclonal ACPA clones 1325:01B09 and 1325:04C03 bind to synovial targets in patients with RA but not in healthy controls. The immunohistochemistry images illustrate binding patterns of the monoclonal ACPA clones 1325:01B09 and 1325:04C03 to synovial tissues obtained from healthy donors (A) or patients with RA (B). Mouse IgG1 and the non-citrulline-specific 1362:01E02 (E02) monoclonal antibody were used as controls. CD55 and podoplanin (PDPN) stainings highlight FLS-rich areas of the synovial membrane. Immunohistochemistry stainings were analysed with light microscopy using 250x original magnification. (C) Confocal microscopy images illustrate a partial colocalisation of the ACPA clone 1325:01B09 with CD55 (C) and PDPN (D), highlighted by closed arrows. Stainings with the monoclonal ACPA 1325:04C03 and the control 1362:01E02 antibodies are also shown. Moreover, partially colocalisation of 1325:01B09 F(ab’)2 with PDPN were highlighted by arrows (E). An original magnification of 400x was used for confocal microscopy. ACPA, anticitrullinated protein/peptide antibody; DAPI, 4,6-diamidino-2-phenylindole; FLS, fibroblast-like synoviocytes; RA, rheumatoid arthritis.

We further investigated cellular binding patterns in synovial biopsies obtained from both healthy volunteers and patients with RA. None of the ACPA clones labelled the uninflamed synovial tissues (figure 6A). In contrast, we detected 1325:01B09 binding to multiple cells from the dense layer of CD55 and podoplanin-(PDPN)-positive fibroblasts in the synovial membrane of two out of the four tested inflamed RA synovial tissues, whereas virtually no binding of the clone 1325:04C03 was observed in the CD55 and/or PDPN-positive areas (figure 6B). No correlation was seen between the staining pattern and disease characteristics. The co-localisation of 1325:01B09 binding sites with CD55-positive (figure 6C) and PDPN-positive FLS (figure 6D), as well as co-localisation of the F(ab’)2 1325:01B09 fragments with PDPN-positive FLS (figure 6E) was further confirmed in two additional inflamed RA synovial tissue using confocal microscopy.

## Discussion

In this report, we describe that steady-state FLS are unresponsive to ACPAs but can be rendered sensitive to these autoantibodies in the presence of additional stimuli. A key event in priming synovial fibroblasts to ACPA is an inducible PAD-2 and PAD-4 expression that is accompanied by increased protein citrullination. Importantly, individual monoclonal ACPAs exhibited different capacities to induce migration of challenged FLS and activate OC. We provide novel insights into how different ACPA clones in concert might contribute to distinct pathogenic events in the RA development. Furthermore, PAD inhibition may be beneficial in targeting FLS during the development of ACPA-positive arthritis.

We demonstrated that polyclonal ACPA IgGs are able to induce migration and adhesiveness of challenged fibroblasts, independent of their tissue origin. This poses challenges in understanding the specific targeting of the synovial tissue in RA. While tissue-specific antibody-induced vascular changes^26^ and site-specific differences in the responses to pro-inflammatory cytokine priming in skin versus synovial fibroblasts^27^ might play a role, further research is needed to clarify this issue. The lack of effects of polyclonal ACPAs on steady-state fibroblasts is in accordance with the clinical observation that ACPAs alone are not sufficient for development of arthritis in seropositive individuals^28^ and the fact that ACPA infusion in mice does not induce arthritis.^11^ In contrast, challenging by transient cellular stress or exposure to pro-inflammatory signals render the FLS sensitive to ACPA effects. Furthermore, while not having a direct effect on FLS, RF is able to potentiate the ACPAs effects on the FcγR-negative FLS, potentially by forming complexes with the cell bound ACPAs and consequently recruiting more of the activated surface molecules, which are engaged in ACPA interactions, as well as their associated signalling components.^29–33^ Our findings provide support to the concept of a stepwise involvement of several molecular mechanisms in ACPA-mediated synovial pathology. Transient synovial events such as trauma^34–38^ or viral infections,^39 40^ which often cause joint inflammation and have been associated with increased levels of pro-inflammatory cytokines and chemokines, including IL-8, as well as concomitant presence of the RF may contribute to synovial fibroblast sensitisation. A majority but not all individual polyclonal ACPA preparations show a similar migration promoting effect with the polyclonal ACPA pool, suggesting that additional mechanisms than the ones presented in the current study might be active in a minority of ACPA-positive RA and certainly in a majority of ACPA-negative RA. This remain to be further investigated.

In contrast to OCs, no others tested RA-derived antibodies but ACPAs had an effect on FLS migration, suggesting a citrullination dependency of the observed effect. As such, the paucity of citrullinated targets observed on steady-state FLS might explain the lack of ACPA effects in unchallenged conditions. Indeed, FLS that were challenged by either cellular stress or certain pro-inflammatory signals increased their expression of both PAD enzymes and citrullinated proteins, which sensitised the cells to ACPA effects. Cellular citrullination appears to be important for the ACPA-mediated FLS mobility, as PAD inhibition completely abolished this effect. While other cellular effects of these inhibitors cannot be completely excluded, the blocking experiments with soluble citrullinated proteins and the reproducibility of the effects when using the F(ab’)2 ACPA fragments further strengthens this observation. Importantly, MDA modification, acetylation and homo-citrullination do not appear to influence FLS migration, as demonstrated by the lack of effect of anti-MDA antibodies, the lack of influence of increased or decreased acetylation on the ACPA effects and the migration promoting effects of both homo-citrullinated cross-reactive and non-cross-reactive monoclonal ACPAs.

While citrullination appears to be central for the ACPA effects on FLS, precise identification of the exact cellular target(s) is still a challenge. Although citrullinated fibrinogen has been previously proposed as an interstitial tissue target able to generate immune complexes in the presence of ACPA,^41^ our current findings suggest an additional mechanism where ACPAs might interact with surface cellular targets. As individual ACPAs are cross-reactive with a wide range of citrullinated peptides and proteins through their consensus motifs,^4^ as also confirmed by our blocking experiments, it remains to be determined which of these antigens are relevant for the observed effect. It has been suggested that ACPAs are prone to bind minute amounts of LPS that might interfere with interpretation of the functional effects observed with different ACPAs preparations.^17^ However, our LPS tests were negative and addition of different amounts of exogenous LPS to the FLS cultures with or without ACPAs had no effect on FLS migration (online supplementary figure 9).

10.1136/annrheumdis-2018-214967.supp10 Supplementary data

FLS motility is governed by complex mechanisms, involving a large array of intracellular signalling pathways^42–48^ that mediate consecutive phases of this process: adhesion, migration and invasion, with PI3K and MAPK playing central roles. We describe here a role for PI3K/Akt but not for MAPK phosphorylation in regulating ACPA-induced migration of challenged FLS. PI3K modulates organisation of actin cytoskeleton and lamellipodium formation via Akt phosphorylation, which can result in cytoskeleton remodelling and increased motility.^49^

A central finding of the current study resides in the selective cellular modulation by distinct monoclonal ACPAs. We show a selective increase of OC formation with the 1325:04C03 clone. This is in line with our previous reported increase of OC formation seen with the 1325:04C03 clone,^4^ that now reaches statistical significance at doses as low as 1 µg/mL, due to the larger number of replicates and the lower variability among the OC precursor cells donors. In spite of the typical cross-reactivity of ACPAs, this selective regulation suggests the targeting of non-overlapping cellular autoantigens. The lack of Fcγ receptors on FLS, the identical IgG1 Fc regions in ACPA clones with different functional effects and the positive effect of ACPA F(ab’)2 fragments on FLS migration suggest a role for specific-antigen binding in the observed ACPA effect. We propose that unique ACPA clones/specificities might be responsible for specific pathological features in distinct stages of the development of ACPA+RA and some of these effects might be dependent on additional stress/inflammatory signals. Consequently, as ACPA fine-specificities are subjected to constant evolution and different ACPA combinations are present in different individuals,^50^ the selective effects of individual ACPA clones could contribute to variability in the timing of arthritis development and in the symptoms present in seropositive individuals. Notably, this proposed role of ACPA is fully compatible with potential roles also of antibodies with other specificities in the generation of the various symptoms occurring before and during the course of RA.

In conclusion, we describe a novel potential pathogenic role for ACPAs in FLS migration. Our results also give further support for studies to investigate the early therapeutic and potentially preventive effect of drugs such as PAD enzymes inhibitors that affect the currently described mechanisms of FLS migration.
