# Supplementary material for: Anticitrullinated protein antibodies facilitate migration of synovial tissue-derived fibroblasts
Source: Ann Rheum Dis. 2019 Sep 3;78(12):1621–31. doi: 10.1136/annrheumdis-2018-214967 (PMC6900251; doi:10.1136/annrheumdis-2018-214967)
Supplement: Supplementary data [file annrheumdis-2018-214967supp001.pdf]

**Supplementary file 1**

Material and methods

***Patient demographics***

| | n | Diagnosis | Age Median (Range) | Sex (Female) n (%) | ACPA Status n (%) |
|---|---|---|---|---|---|
| Synovial Fibroblast | 22 | RA | 66 (32-82) | 12 (54.5) | 12(54.5) |
| | 1 | OA | 65 | 1 (100) | 0 (0) |
| Synovial biopsy | 6 | Early RA | 61.5 (48–78) | 3 (50) | 4 (100) |
| | 4 | Healthy | 27.5 (21–58) | 2 (50) | 0 (0) |
| ACPA Pool (2013) | 36 | RA | 61.5 (29–86) | 25 (69.4) | 36 (100) |
| ACPA Pool (2016) | 101 | RA | 63 (27–83) | 78 (77.2) | 101 (100) |
| Individual ACPA Fractions* | 4 | RA | 68.5 (60–84) | 3 (75) | 4 (100) |
| PR3 Ab | 1 | Vasculitis | 31 | 1 (100) | 0 (0) |
| Monoclonal Antibodies | | | | | |
| Anti-CCP Ab | 4 | RA | 54.5 (37–66) | 3 (75) | 3 (75) |
| Anti-MDA Ab | 3 | RA | 68 (66–69) | 2 (66.7) | 2 (66.7) |

* Three of the individual sera are part of the original ACPA pool but sera for production of the individual preparations were sampled at a different point in time than the sera originally used in the production of the ACPA pool. The fourth individual serum is from a patient that was not included in the original ACPA pool.

***Individual sera CCP and RF reactivity***

| Sample | anti CPP2 (Au/ml) | Rheumatoid Factor |
|---|---|---|
| 189 | > 3200 | + |
| 192 | > 3200 | Low |
| 194 | > 3200 | - |
| 198 | 77 | - |

***Monoclonal antibody reactivity***

| Antibody | CCP2 reactivity* (Au/ml at 5ug/ml) | Anti-MDA reactivity** | Anti-Carbamylation reactivity | Acetylated Histone 2B/4*** | validated with alternative methods | | |
|---|---|---|---|---|---|---|---|
| | | | | | Citrullinated Protein western blots | Citrullinated peptide ELISAs ^ | Citrullinated multiplex peptide array |
| 1325:05C06 hIgG1 | 2790 | - | + | + | + | + | + |
| 1325:07E07 hIgG1 | 670 | - | - | - | + | + | + |
| 1325:01B09 hIgG1 | 1455 | - | + | + | + | + | + |
| 1325:04C03 hIgG1 | 1170 | - | - | - | + | + | + |
| 14CFCT2D09 hIgG1 | 310 | - | - | - | + | + | + |
| 14CFCT2H12 hIgG1 | 2400 | - | - | - | + | + | + |
| 37CEPT2C04 hIgG1 | 3010 | - | + | + | + | + | + |
| 1362:01E02 hIgG1 | 0 | - | - | - | - | - | - |
| 146+:01G07 hIgG1 | 0 | + | - | - | n.d. | - | - |
| 1276:01F04 hIgG1 | 0 | + | - | - | n.d. | - | - |
| 1362:03H05 hIgG1 | 0 | + | - | - | n.d. | - | - |

* cut off for positivity is 25 (CCPlus, ELISA, EuroDiagnostica AB)
** in-house protein ELISA (MDA-modified BSA), mAbs tested at 5µg/ml
*** in-house peptide ELISA, mAbs tested at 5µg/ml
^ All ACPAs were validated for citrulline-reactivity by long titrations, with reactivity detected down to 5-80 ng/mL
n.d. not done

Table shows the reactivity of the monoclonal antibodies against various post translational modification. All ACPAs are highly mutated and have Fab-glycosylation sites.

Heat map reactivity of the monoclonal antibodies by CCP2 ELISA and ISAC.

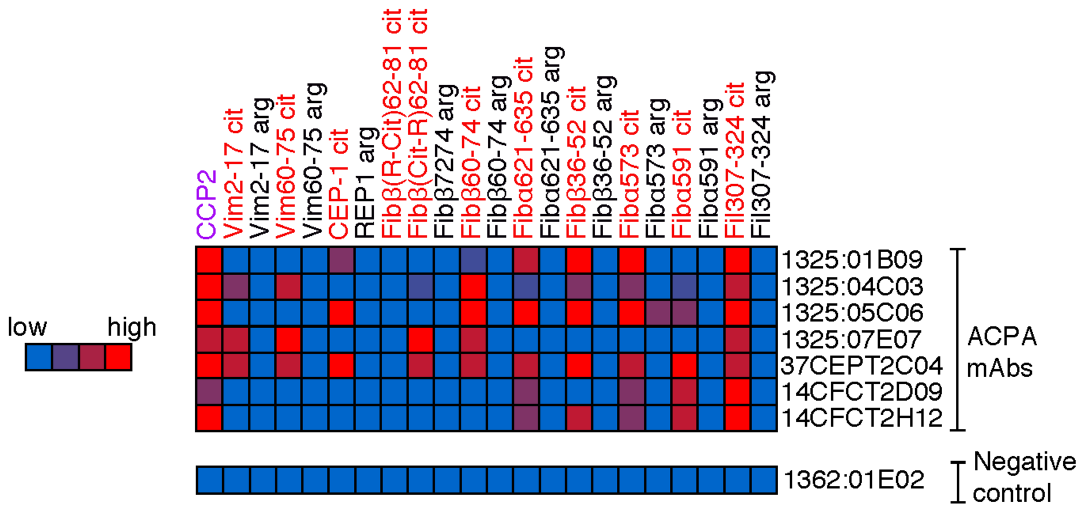

*Endotoxin (the LAL test and the THP-1-Xblue-MD2/CD14 assay), aggregation and rheumatoid factor measurement*

| Antibody | Aggreagation Test (Monomer %) | Endotoxin LAL Test (EU/mL) | THP-1-Xblue-MD2/CD14 assay (EU/mg) | RF Test (at 1 µg/mL) | |
|---|---|---|---|---|---|
| | | | | IgM (ng/mL) | IgG (ng/mL) |
| Polyclonal anti-CCP2 hIgG | 92 | < 0.05 | < 0.5 | 0.50 (< LoD) | 0.23 (< LoD) |
| Polyclonal ACPA Fab2 | - | - | < 0.5 | - | - |
| Flowthrough IgG | 96 | < 0.05 | < 0.5 | 0.08 (< LoD) | 0.20 (< LoD) |
| Flowthrough Fab2 | - | - | < 0.5 | - | - |
| Pat_189 anti-CCP2 hIgG | 88 | < 0.05 | - | 0.08 (< LoD) | 0 (< LoD) |
| Pat_189 Flowthrough hIgG | 99 | < 0.05 | - | 0.22 (< LoD) | 0 (< LoD) |
| Pat_192 anti-CCP2 hIgG | 96 | < 0.05 | - | - | - |
| Pat_192 Flowthrough hIgG | 99 | < 0.05 | - | - | - |
| Pat_194 anti-CCP2 hIgG | 91 | < 0.05 | - | - | - |
| Pat_194 Flowthrough hIgG | 99 | < 0.05 | - | - | - |
| Pat_198 anti-CCP2 hIgG | 73 | < 0.05 | - | - | - |
| Pat_198 Flowthrough hIgG | 98 | < 0.05 | - | - | - |
| 1325:05C06 hIgG1 | 99 | < 0.05 | < 0.5 | - | - |
| 1325:07E07 hIgG1 | 98 | < 0.05 | < 0.5 | - | - |
| 37T+:02C04 hIgG1 | 99 | < 0.05 | < 0.5 | - | - |
| 14T+:02D09 hIgG1 | 97 | 0.06 | < 0.5 | - | - |
| 14T+:02H12 hIgG1 | 99 | < 0.05 | < 0.5 | - | - |
| 1325:01B09 hIgG1 | > 90 | < 0.05 | < 0.5 | - | - |
| 1325:01B09 Fab2 | - | - | < 0.5 | - | - |
| 1325:04C03 hIgG1 | 98 | 0.04 | < 0.5 | - | - |
| 1325:04C03 Fab2 | - | - | < 0.5 | - | - |
| 1362:01E02 hIgG1 | > 90 | < 0.05 | < 0.5 | - | - |
| 1362:01E02 Fab2 | - | - | < 0.5 | - | - |
| 146+:01G07 hIgG1 | 99 | < 0.05 | < 0.5 | - | - |
| 1276:01F04 hIgG1 | 90 | < 0.05 | < 0.5 | - | - |
| 1362:03H05 hIgG1 | > 90 | < 0.05 | < 0.5 | - | - |
| PR3 hIgG | 94 | 0.360 | - | - | - |
| PR3 Flowthrough hIgG | 96 | 0.102 | - | - | - |

The antibody reagents used in the different cell assays were tested for the presence of endotoxin at a concentration of 10 µg/mL by the limulus amebocyte lysate (LAL) test at the Karolinska University laboratory according to the routine protocol. Endotoxin levels were also analyzed by using the THP1-XBlue-MD2-CD14 cell line (Invivogen, Toulouse, France). The cells were incubated in the presence of 50mg/ml antibodies at the density of 0.5x106 cells/ml for 24 hours. Ultrapure E. Coli K12 LPS (Invivogen, Toulouse, France) was diluted to assess assay sensitivity. These cells react to TLR stimulation by producing secreted embryonic alkaline phosphatase, which was quantified using the QUANTI-Blue dye (Invivogen) according to the manufacturer's recommendations.

Aggregation test was performed by high-resolution protein separation using ENrich™ high-resolution SEC 650 (Bio-Rad, Sweden) size exclusion column with ÄKTAexplorer (GE Healthcare Life Sciences, Sweden). Briefly, 10 µg of the sample is run at a flow rate of 1ml/min. The proportion of monomeric IgG is calculated by estimating the area under the curve for monomeric IgG which has a retention time of approx. 12.5 ml. RF isotypes IgM and IgG were analyzed with EliA™ (Phadia AB, Uppsala, Sweden), according to the manufacturer's

instructions. Briefly, all samples were diluted to a concentration of 1 µg/mL in the kit sample diluent and then assayed in duplicates. Results were converted from IU/mL to µg/mL by the manufacturer. The limit of detection (LoD) for the IgM and IgG RF assays are 1.82 and 0.50 ng/mL respectively.

***Cell cultures***

FLS were isolated from synovial tissues (knee=15, hip=7) through enzymatic digestion using 4mg/ml Collagenase A and 0.1mg/ml DNase I (Roche, Mannheim, Germany). The dissociated cells were cultured in Dulbecco's Modified Eagle Medium (DMEM) complemented with heat-inactivated fetal bovine serum (FBS), 100 U/mL penicillin, 100 µg/mL streptomycin and 2mM L-glutamine (all from Sigma-Aldrich, St. Louis, MO, USA). Non-adherent cells were removed after overnight incubation and new medium was added. The cells were detached with Trypsin-EDTA (Sigma-Aldrich) and split in new medium 1:2 every time when reaching approximately 80% confluence.

Human dermal fibroblast (HDF) and osteoarthritis synovial fibroblast (OASFs) were cultured with DMEM complemented with 10% heat-inactivated fetal bovine serum (FBS), 100 U/mL penicillin, 100 µg/mL streptomycin and 2mM L-glutamine (all from Sigma-Aldrich, St. Louis, MO, USA).

***LPS Titration***

LPS with a broad range of concentrations from 50ng/ml to 0.125ng/ml (7.5EU/ml to 0.02EU/ml) were tested on FLS migration in the presence or absence of 1µg/ml ACPA and control IgG. FLS migration were assessed by IncuCyte Zoom live cell imaging system.

***Citrullination of proteins***

Citrullination of the fibrinogen, enolase, histone h4 (Cayman chemicals, Michigan, USA) and vimentin (Peprotech, Stockholm, Sweden) was performed using 2 U/mg of rabbit skeletal

muscle PAD enzyme (Sigma-Aldrich, Stockholm, Sweden) or human PAD-2 enzyme (Cayman chemicals, Michigan, USA) in buffer containing 100 mM Tris, 10 mM $CaCl_2$, 5 mM dithiothreitol (DTT) for 2 h at 37 °C (pH 7.6) The reaction was stopped by the addition of 20 mM EDTA.

***F(ab')2 generation***

F(ab')2 ACPAs and control IgGs were generated using a commercially available kit (Genovis, Lund, Sweden). Briefly, antibodies are incubated in a column immobilized with cysteine protease enzyme that digests below the hinge region, generating a pool of F(ab')2 and Fc fragments. Followed by the incubation with the capture select columns with affinity towards the Fc fragments allowing the F(ab')2 fragments generation. All the F(ab')2 tested in assays are used in molar concentrations equivalent to original antibodies. Both whole ACPAs and F(ab’)2 ACPAs were loaded and electrophoresed on 4-20% SDS-PAGE (Bio-Rad, Solna, Sweden), followed by InstantBlue (Expedeon, Swavesey, UK) or coommassie blue staining for purification check.

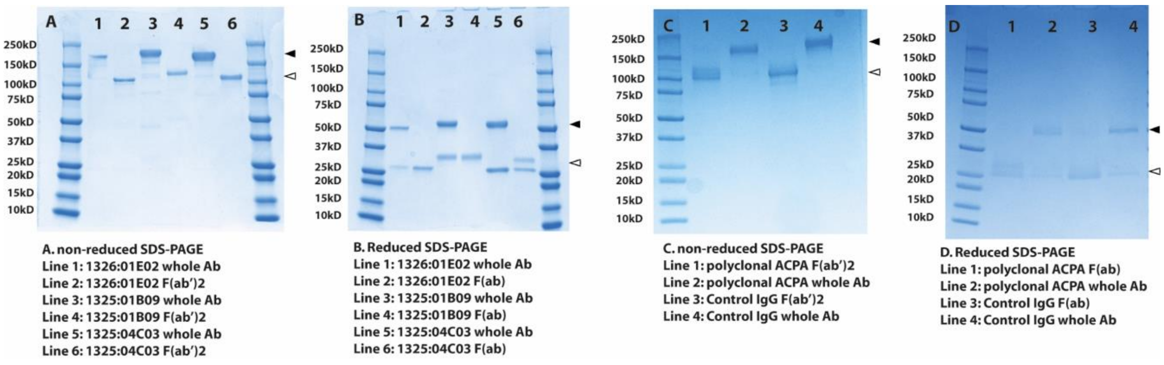


Closed arrows indicate Fc fragments in all gel.

Open arrows indicate F(ab')2 fragments in non-reduce gel (A and C) and F(ab) fragments in reduced gel (B and D).

ACPA mAbs have different N-glycosylation sites would explain the differences in size observed.

***F(ab')2 labelling***

Alexa Fluor 594 microscale protein labelling kit (A30008) (Invitrogen detection technologies, Stockholm, Sweden) was used for labelling the F(ab')2 fragments of 1325:04C03, 1325:01B09 and 1362:01E02 according to manufacturer instructions.

***Immunoblotting***

Cells were lysed in loading buffer (62.5mM Tris-HCl pH 6.8, 25% glycerol, 2% SDS and 0.01% Bromophenol Blue) and aliquots corresponding to $1.5x10^4$ cells were loaded in each lane in an SDS-PAGE gel (Bio-Rad, Solna, Sweden). Proteins were transferred to nitrocellulose membranes, which were then blocked with 5% Blotting-Grade Blocker (Bio-Rad, Solna, Sweden) or 5% BSA (Merch, German) in TBST (0.01µM sodium phosphate buffer, 0.5µM NaCl and 0.05% Tween 20, pH 7.3) buffer for 30 minuets at room temperature. Membranes were incubated with primary antibodies overnight at 4°C, then washed 15 mins three times in TBST buffer and incubated with peroxidase-conjugated secondary antibodies at room temperature for 1 hours. Signals were detected using SuperSignal West Femto Maximum Sensitivity Substrate (Thermo Fisher Scientific, USA) and developed with either with film (themofisher scientific, Sweden) or chemidoc image system (Bio-Rad, USA). Band intensities were determined using Image Lab 5.2.1 Software (Bio-Rad).

| Antibody used in Immunoblotting | Dilution | Company |
|---|---|---|
| Phospho-Akt (Thr308) (D25E6) XP Rabbit mAb | 1:1000 | Cell Signaling Technology |
| Phospho-Akt (Ser473) (D9E) XP Rabbit mAb | 1:2000 | Cell Signaling Technology |
| Phospho-SAPK/JNK (Thr183/Tyr185) (G9) Mouse mAb | 1:2000 | Cell Signaling Technology |
| Phospho-p38 MAPK (Thr180/Tyr182) (28B10) Mouse mAb | 1:2000 | Cell Signaling Technology |
| Phospho-p44/42 MAPK (Erk1) (Tyr204)/(Erk2) (Tyr187) (D1H6G) Mouse mAb | 1:1000 | Cell Signaling Technology |
| GAPDH Loading Control Monoclonal Antibody (GA1R) | 1:5000 | Thermo Fisher Scientific |
| Acetyl-Histone H4 (Lys8) Antibody | 1:1000 | Cell signaling Technology |

***Immunhistochemistry***

Synovial fibroblasts were stained on 8-well chamber slides (Thermo Fisher Scientific, US). Cryostat sections of synovial biopsies with a thickness of 7μm were prepared from samples obtained from healthy donors (n=4) and RA patients (n=4) undergoing hip (n=3) or knee (n=5) arthroplasty. Slides were fixed using 2% methanol-free formaldehyde (Thermo Fisher Scientific, US) at $4^{\circ}$C for 10 minutes and then washed with PBS. Slides were then air dried and stored at -20°C until use.

cells and tissues were permeabilized with PBS/saponin (0.1%, pH7.4) for intercellular staining or kept non-permeabilized for surface staining. After blocking of endogenous peroxidase with 2% $H_2O_2$, avidin-biotin activity (Avidin/Biotin blocking kit, Vector Laboratories, UK) and/or Fc receptors (human BD Fc block, BD bioscience, US), the cells and tissues were stained with primary antibodies followed by HRP-conjugated antibodies as secondary antibody. The slides were developed by Vectastain Elite ABC and DAB (Vector Laboratories, UK) and then counterstained with Mayer's hematoxylin. Images were analyzed using light microscope (Reichert Polyvar 2 type 302001, Leica, German).

| Antibody used in ICC | Concentration (µg/ml) | Company |
|---|---|---|
| biotinylated polyclonal ACPA IgGs | 5 | Purified from RA Patients |
| biotinylated non-ACPA control IgGs | 5 | Purified from RA Patients |
| biotinylated monoclonal ACPA clone 1325:01B09 | 5 | recombinant antibody |
| biotinylated monoclonal ACPA clone 1325:04C03 | 5 | recombinant antibody |
| biotinylated monoclonal ACPA clone 1362:01E02 | 5 | recombinant antibody |
| Anti-CXCR1 (ab10400) | 5 | Abcam |
| anti-CXCR2 (ab24963) | 10 | Abcam |
| Podoplanin Monoclonal Antibody (NZ-1.3) | 2 | ThemoFisher Scientific |
| Human BD Fc Block | 5 | BD Bioscience |

***Immunofluorescence and intensity quantification***

Synovial fibroblasts were stained on 8-well chamber slides (Thermo Fisher Scientific, US). Cryostat sections of synovial biopsies with a thickness of 7µm were prepared from samples RA patients (n=2) undergoing joint arthroplasty. Cells and tissues were kept non-permeabilized. After 5% BSA and Fc receptors blocking by BD human Fc block in PBS, the slides were incubated with primary antibody (at 4 °C overnight) followed by washing with TPBS for 3 times 45 minuets in total. Slieds were then incubated with secondary antibodies (1hour at RT) followed by washing with TPBS 3 times 45 minutes in total and then counterstained with 4,6-diamidino-2-phenylindole (DAPI). Samples were analyzed using Leica TCS SP laser scanning confocal microscope. Mean intensities of PAD2, PAD4 and ACPA in individual cells were determined using Image Lab 5.2.1 Software (Bio-Rad).

| Antibody used in IF | Concentration (µg/ml) | Company |
|---|---|---|
| anti-CD55 (MCA1614) | 1 | Serotec |
| anti-PAD2 | 0.2 | Cosmo |
| anti-PAD4 | 2 | abcom |
| Podoplanin Monoclonal Antibody (NZ-1.3) | 2 | ThemoFisher Scientific |
| biotinylated 1325:01B09 | 5 | recombinant antibody |
| biotinylated 1325:04C03 | 5 | recombinant antibody |
| biotinylated 1362:01E02 | 5 | recombinant antibody |
| 1325:01B09 F(ab')2 alexa 594 | 5 | recombinant antibody |
| 1325:04C03 F(ab')2 alexa 594 | 5 | recombinant antibody |
| 1362:01E02 F(ab')2 alexa 594 | 5 | recombinant antibody |
| biotinylated polyclonal ACPA | 10 | Purified from RA Patients |
| Goat anti-Mouse Alexa Fluor 594 | 2 | Life technologies |
| Goat anti-Rabbit Alexa Flour 488 | 2 | Life technologies |
| Goat anti-rat Alexa Flour 488 | 2 | invitrogen |
| Streptavidin Alexa Fluor 488 | 2 | Life technologies |
| Human BD Fc Block | 5 | BD bioscience |

***Adhesion assay***

Adhesion assay was performed on 16 well E-plate (ACEA Biosciences, US) and monitored by xCELLigence System Real-Time Cell Analyzer (ACEA bioscience, US). After serum starvation, SFs were suspended in medium containing 2% FBS and plated using the density of 2500 cells/well. Polyclonal ACPA and non-ACPA control IgG were added with concentration of 1μg/ml. Impedance-based signals were monitored every minute from 0 to 1 hour, every 5 minutes up to 13 hours and every 15 minutes up to 36 hours. Impedance signals were analyzed using xCelligence (version 2.2) and reported as a unit-less parameter called Cell Index (CI), calculated as follows: (impedance at time point 'n' - impedance in the absence of cells)/nominal impedance or as slope, calculated as follows: (ΔCI/ΔT(hour)).

***Flow cytometry***

The cells were collected using non-enzymatic detachment reagent (Sigma-Aldrich), washed in PBS and then stained in PBS, 1%FBS for 45 minutes using antibodies in table below. Dead cells were excluded using the LIVE/DEAD near-IR dead cell stain (ThermoFisher Scientific, Waltham, MA, USA). Fluorescence intensities were measured with FACSVerse flow cytometer (BD Biosciences, San Jose, CA, USA) and analyzed by Flowjo 9.2 software (Flowjo, Ashland, OR, USA)

| **Antibodies (Flow cytometry)** | **Fluorochrome** | **Company** |
|---|---|---|
| Anti-Fibroblast (clone REA165) | FITC | Miltenyi Biotech |
| CD29 | PerCP/Cy5.5 | Biolegend |
| CD32 | PerCP/Cy5.5 | Biolegend |
| CD64 | FITC | Biolegend |
| CD16 | AlexaFluor647 | BD Pharmingen |
| CXCR1 | PE | R&D Systems |
| CXCR2 | APC | R&D Systems |
| REA Control IgG1 | FITC | Miltenyi Biotech |
| Mouse IgG1 | PerCP/Cy5.5 | Biolegend |
| Mouse IgG2b | PerCP/Cy5.5 | Biolegend |
| Mouse IgG1 | FITC | Biolegend |
| Mouse IgG1 | AlexaFluor647 | BD Pharmingen |
| Mouse IgG2a | PE | R&D Systems |
| mouse IgG2a | APC | R&D Systems |

***Mass spectrometry***

Cell pellets were lysed in 100 µl of 5% SDS in 50 mM TEAB (triethylammonium bicarbonate) buffer, pH 7.55. DNA was disrupted using 1U of bensonaze and incubation at 4°C for 30 min. Lysates were clarified by centrifugation at 15 000 x g for 30 min at 4°C and pellets were discarded. Peptide digests were prepared on a S-Trap™ Mini Spin columns (Protifi, Huntington, NY) according to the manufacturer's protocol. Obtained peptides were eluted by 50% acetonitrile (MeCN) in 0.2% formic acid (FA) and dried in speed-vac.

***LC-MS/MS and data analysis***

The concentration of obtained peptides was determined using NanoDrop and equal amounts of starting material were loaded for the LC-MS/MS analysis. LFQ was performed on the basis of retention time, mass and the corresponding intensities throughout all samples (16034 peptides) using Bruker software. Samples were suspended in loading buffer with 3% MeCN in 0.1% FA and 2 µg of obtained peptide mixtures were loaded onto a Dionex Ultimate 3000 HPLC system (Thermo Fisher Scientific) coupled to an o-QTOF impact II™ (Bruker Daltonics) mass spectrometer equipped with CaptiveSpray ion source (Bruker Daltonics). Peptide samples were trapped on an Acclaim PepMap trap column (C18, 3 µm, 100Å, 75 µm x 20 mm), and separated on a PepMap RSLC column (C18, 2 µm, 100Å, 75 µm x 50 cm, Thermo Fisher Scientific) using a gradient of A (0.1% FA) and B (95% MeCN, 0.1% FA), ranging from 3 % to 45 % B in 150 min with a flow of 0.3 µL/min. LC-MS/MS data were acquired using a data-dependent auto-MS/MS method selecting the 20 most abundant precursor ions for fragmentation. The mass range of the MS scan was set from *m/z* 150 to 1600. Dynamic exclusion duration was 0.4 min. All spectra were acquired with internal calibration using sodium formate clusters. Data was analyzed with ProteinScape software (Bruker Daltonics) using the Mascot search engine, (Matrix Science Ltd., www.matrixscience.com). The following parameters were used for the database search:

tryptic digestion (maximum of 2 miscleavages); carbamidomethylation (C) as a fixed modification; oxidation (M), deamination (N/Q) and citrullination (R) as the variable modifications; 10 ppm as the precursor tolerance; and 0.05 Da as the fragment tolerance.

***Cell viability, proliferation and apoptosis assays***

Cells were treated with Cl-amidine, wortmannin and SF-1670 for 2 hours piror to scratching. At the end of experiment, cell-free supernatants were collected. Cell viability were analyzed by Cytotoxicity Detection Kit (LDH) (Roche, Germany), proliferation were evaluated by BrdU Cell Proliferation ELISA Kit (colorimetric) (ab126556, Abcom, Cambridge, UK) and apoptosis were investigated by were measured by annexin V apoptosis and necrosis assay (Promega, Stockholm, Sweden) according to the manufacturer's recommendations.
