# Supplementary figures and images for: Anticitrullinated protein antibodies facilitate migration of synovial tissue-derived fibroblasts
Source: Ann Rheum Dis. 2019 Sep 3;78(12):1621–31. doi: 10.1136/annrheumdis-2018-214967 (PMC6900251; doi:10.1136/annrheumdis-2018-214967)

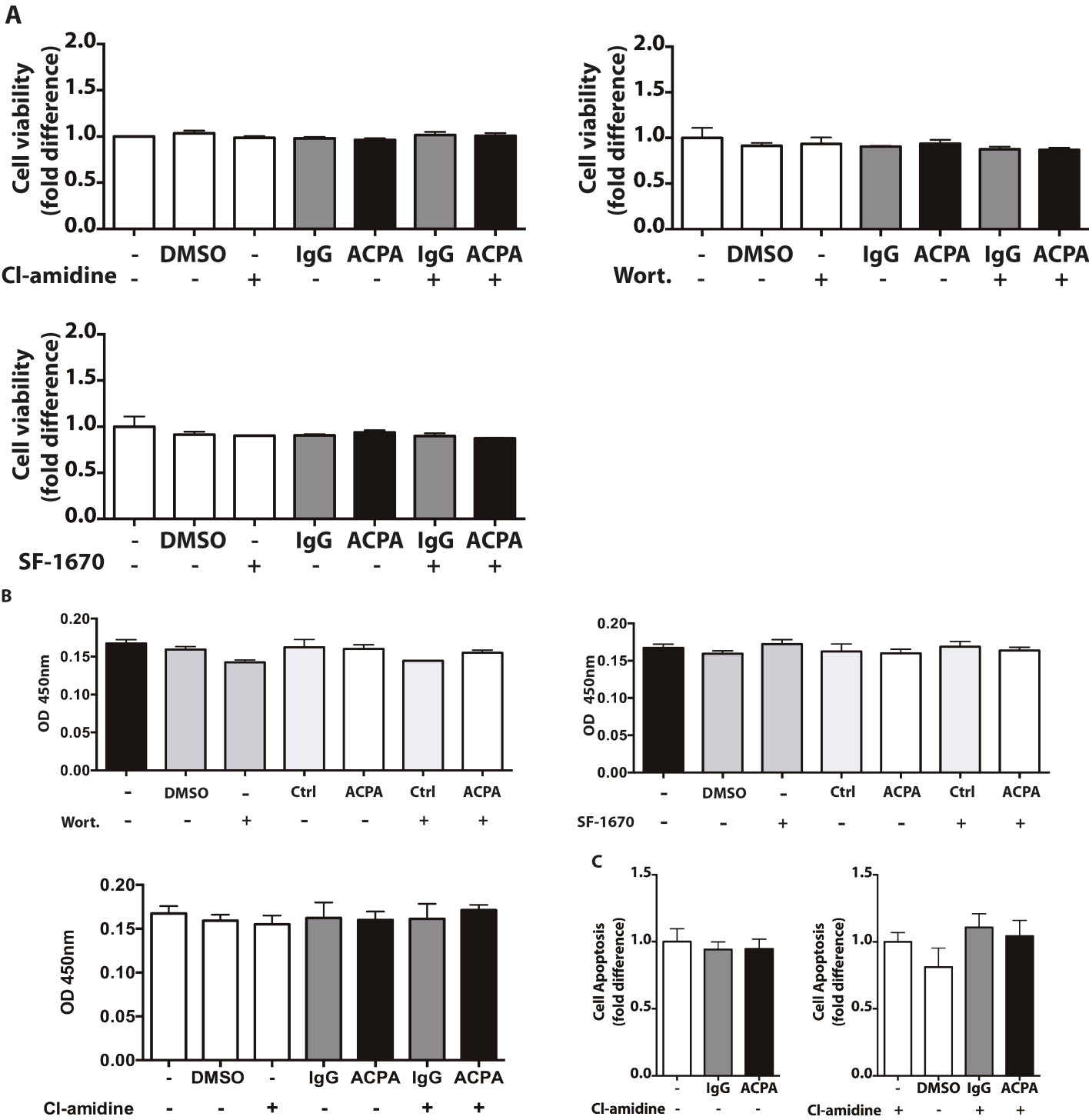

Supplement: Supplementary data [file annrheumdis-2018-214967supp002.pdf]

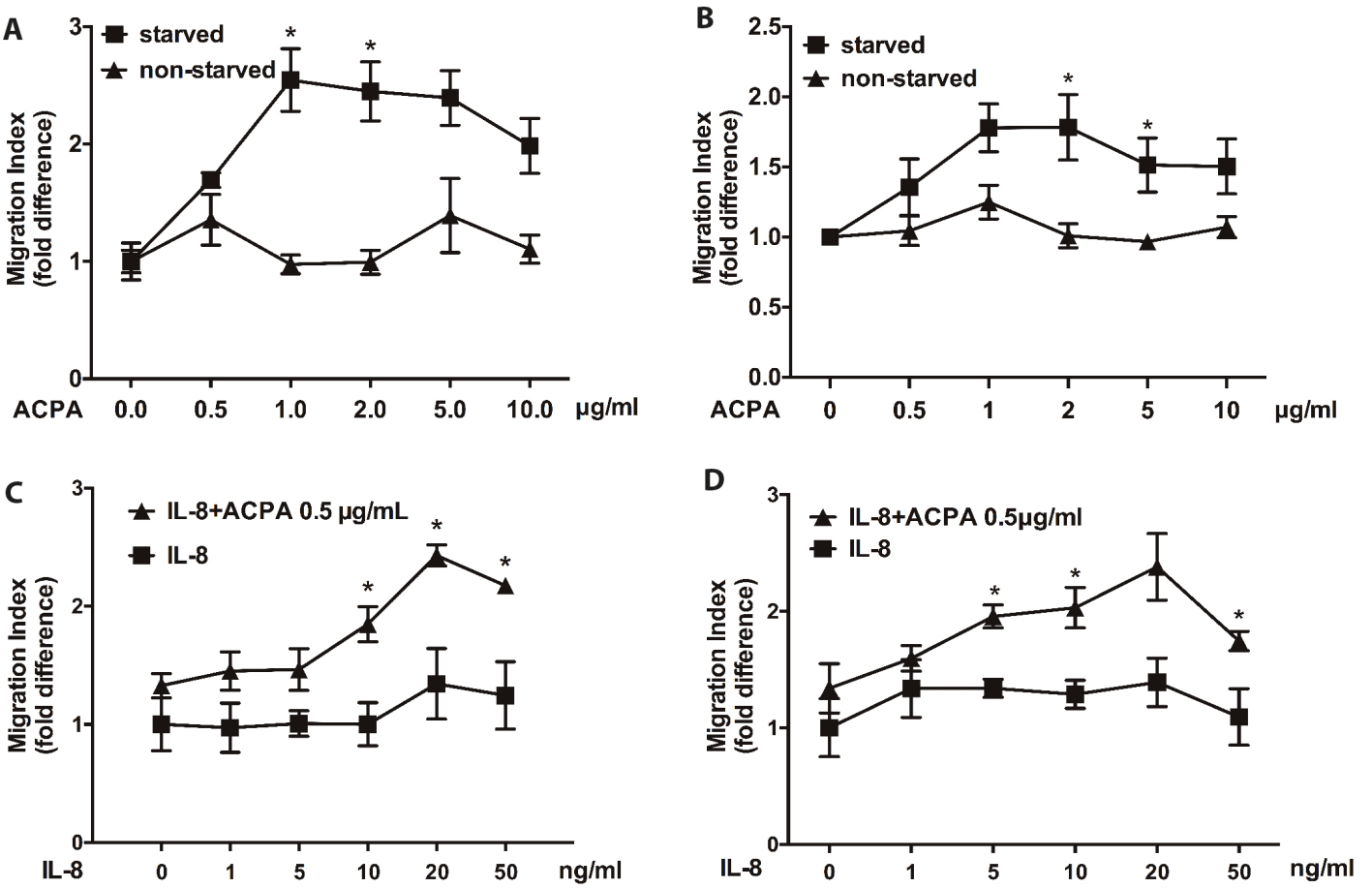

Supplement: Supplementary data [file annrheumdis-2018-214967supp003.pdf]

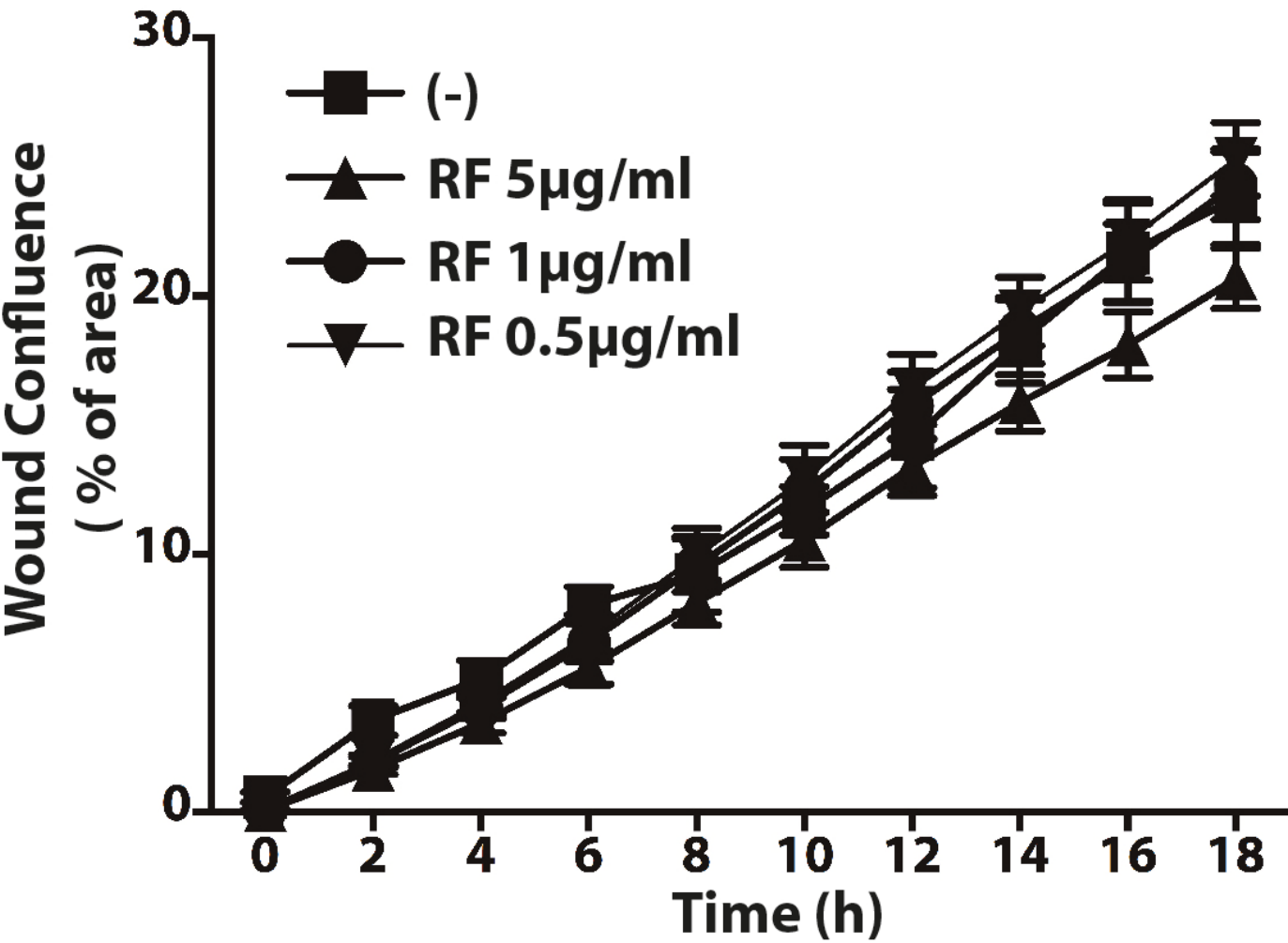

Supplement: Supplementary data [file annrheumdis-2018-214967supp004.pdf]

A. Non-starved

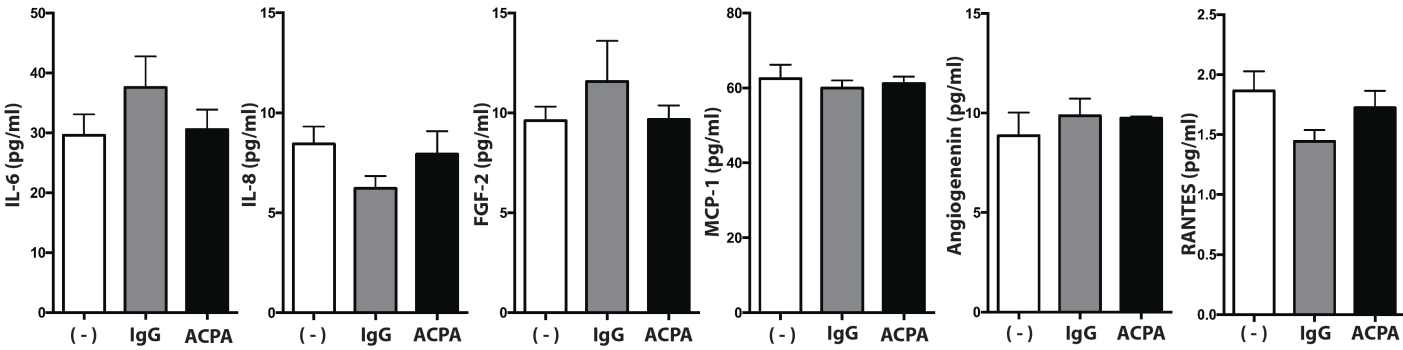

B. Starved

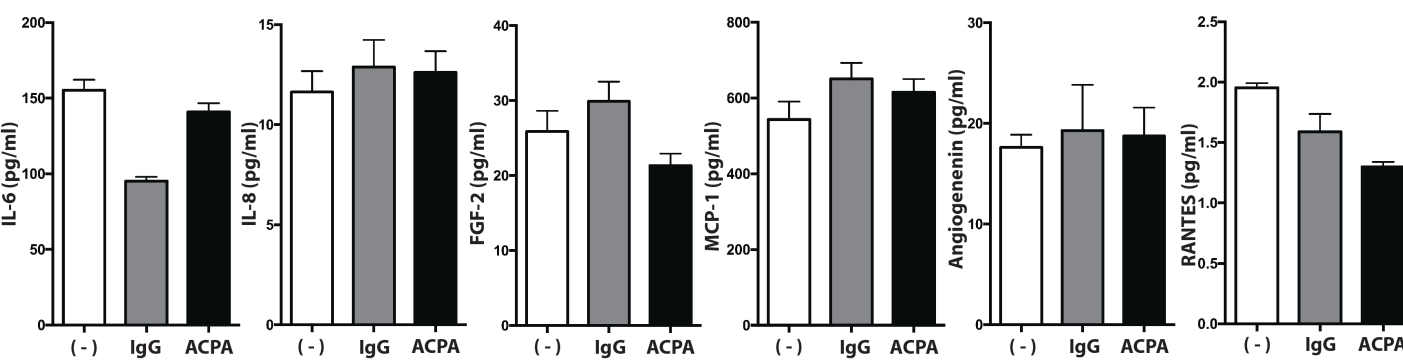

C. MMPs

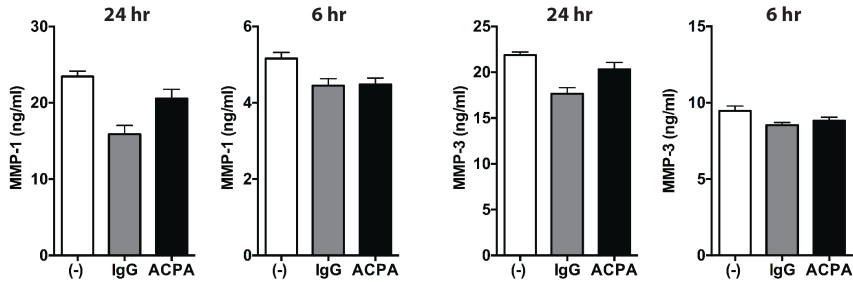

Supplement: Supplementary data [file annrheumdis-2018-214967supp005.pdf]

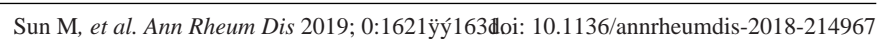

Supplement: Supplementary data [file annrheumdis-2018-214967supp008.pdf]

monoclonal ACPA titration

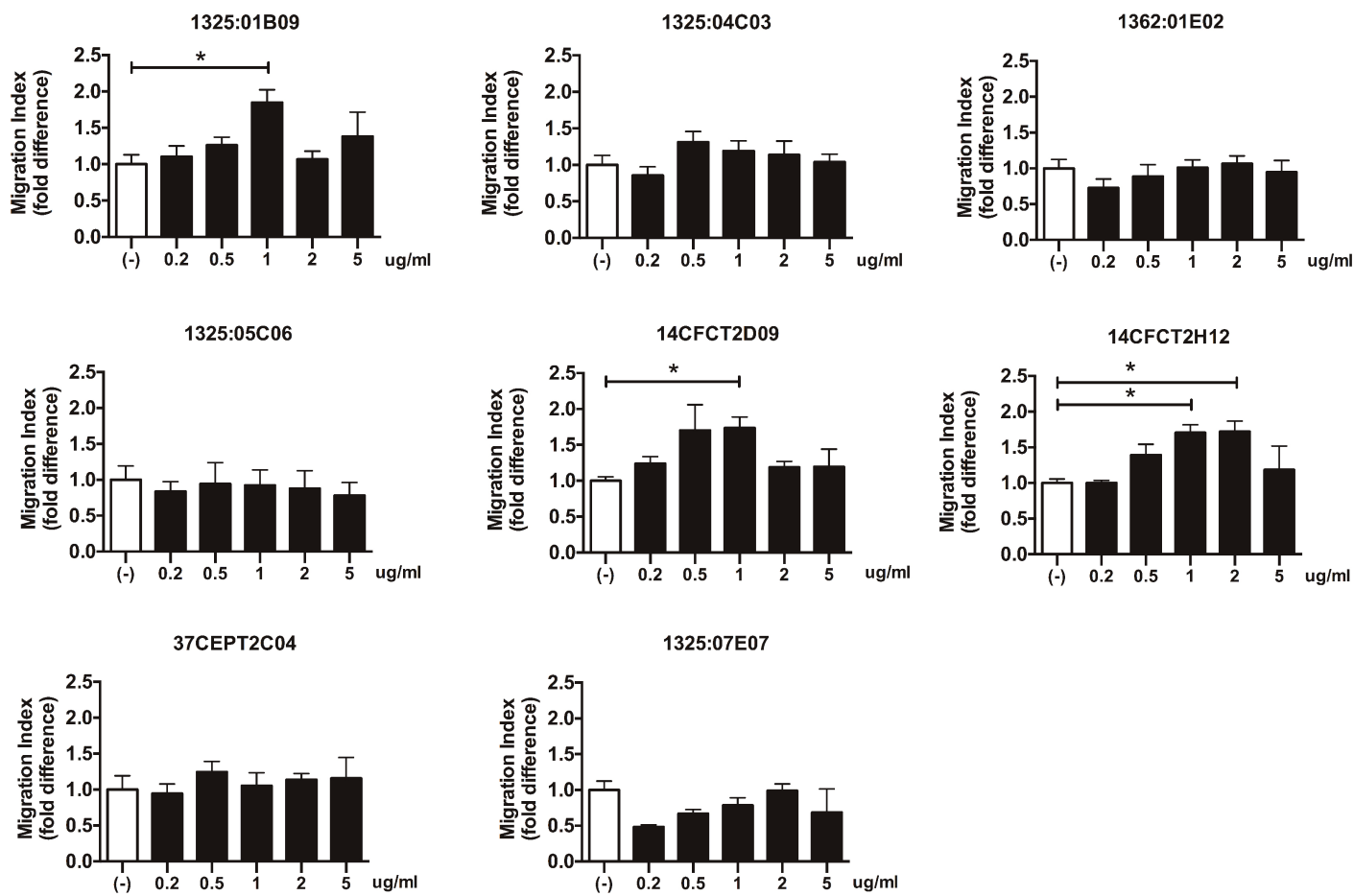

Supplement: Supplementary data [file annrheumdis-2018-214967supp009.pdf]

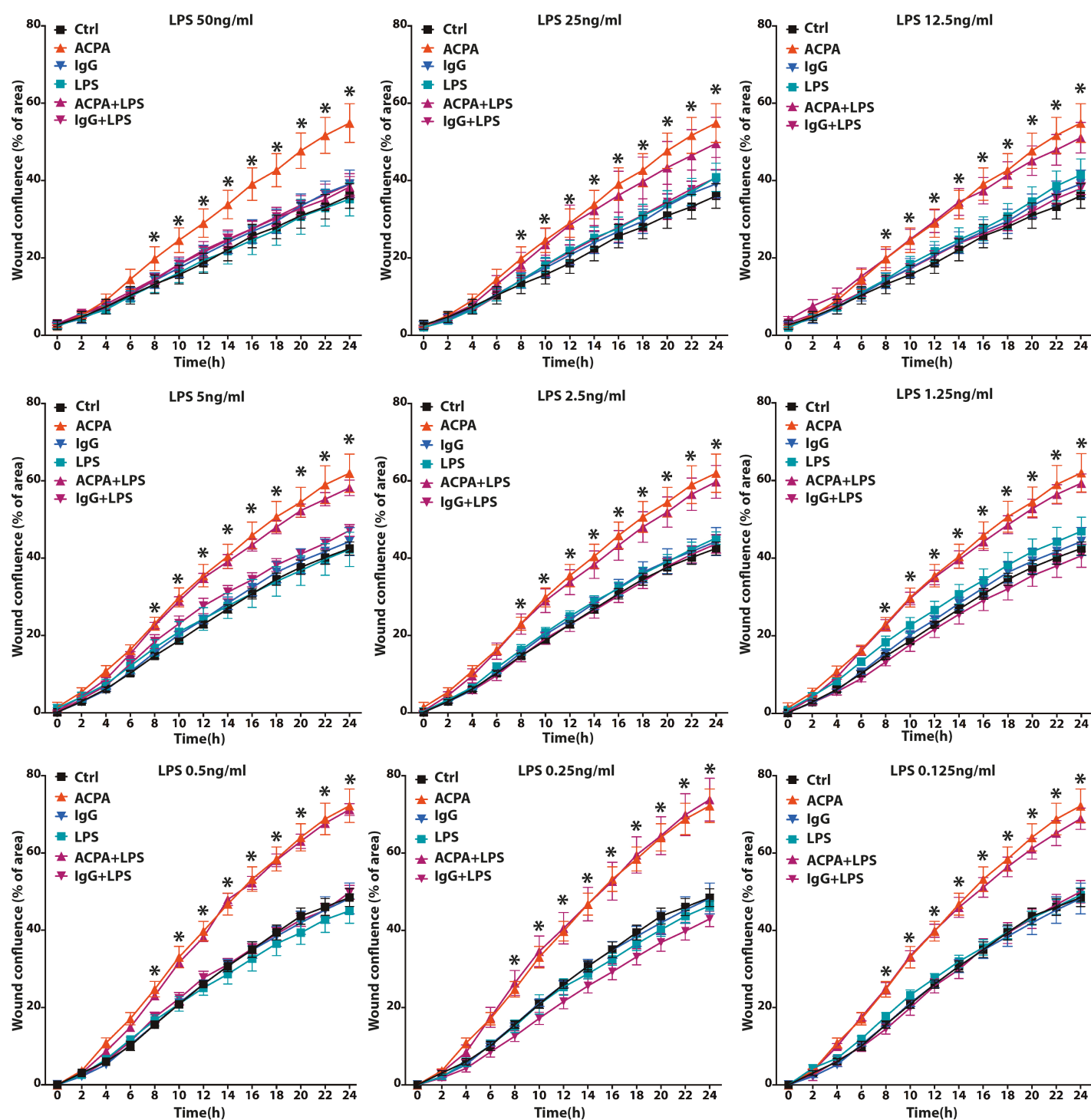

Supplement: Supplementary data [file annrheumdis-2018-214967supp010.pdf]
