# Supplementary material for: Anticitrullinated protein antibodies facilitate migration of synovial tissue-derived fibroblasts
Source: Ann Rheum Dis. 2019 Sep 3;78(12):1621–31. doi: 10.1136/annrheumdis-2018-214967 (PMC6900251; doi:10.1136/annrheumdis-2018-214967)
Supplement: Supplementary data [file annrheumdis-2018-214967supp006.pdf]

Figure 2A		Intensity (Mean ± SD)	P value
PAD2	NC	3.131 ± 5.237	< 0.0001
	Starved	93.21 ± 53.53	
PAD4	NC	0.2729 ± 0.1649	< 0.0001
	Starved	144.3 ± 42.71	
ACPA	NC	5.370 ± 0.9712	< 0.0001
	Starved	143.9 ± 42.46	

Figure 4H and G		Intensity (Mean ± SD)	P value
PAD2	NC	6.352 ± 4.198	-
	Starved	111.7 ± 46.74	< 0.0001
	IL-8	124.0 ± 50.46	< 0.0001
PAD4	NC	4.995 ± 4.536	-
	Starved	152.5 ± 43.81	< 0.0001
	IL-8	130.5 ± 49.16	< 0.0001
ACPA	NC	3.473 ± 2.871	-
	Starved	92.19 ± 48.03	< 0.0001
	IL-8	90.37 ± 46.41	< 0.0001

Each group contains intensity values from 3 representative fields (images) containing at least 150 cells in total.
