# Supplementary material for: Anticitrullinated protein antibodies facilitate migration of synovial tissue-derived fibroblasts
Source: Ann Rheum Dis. 2019 Sep 3;78(12):1621–31. doi: 10.1136/annrheumdis-2018-214967 (PMC6900251; doi:10.1136/annrheumdis-2018-214967)

A

Sample type	Protein ID	Protein Name	Peptide sequense	Score
Non-ststarved	Q14517	Protocadherin Fat 1	R.LSFVTP**Cit**HHR.A	30,2
Starved	Q14517	Protocadherin Fat 1	R.LSFVTP**Cit**HHR.A	31
Starved	O75604	Ubiquitin carboxyl-terminal hydrolase 2	R.NLG**Cit**SPMLARTR.K	39,7
Starved	Q9NZ71	Regulator of telomere elongation helicase 1	R.VCPYYLS**Cit**NLK.Q	20,4
Starved	Q5SRE5	Nucleoporin NUP188 homolog	R.LQSKIEDM**Cit**IK.V	22
Starved	Q13011	Delta(3,5)-Delta(2,4)-dienoyl-CoA isomerase	MAAGIVAS**Cit**RLR.D	20,9
Starved	P57078	Receptor-interacting serine/threonine-protein kinase 4	K.S**Cit**LFDTK.H	34,1
Starved	Q6ZVT0	Inactive polyglycylase TTLL10	R.VVQ**Cit**YIQNPLLVDGRK.F	20,8
Starved	O15020	Spectrin beta chain, non-erythrocytic 2	K.RLEDAL**Cit**AQQFYR.D	29,5

B

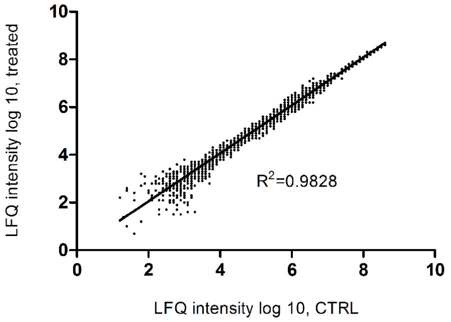

Supplement: Supplementary data [file annrheumdis-2018-214967supp007.pdf]
